# “And the Next Thing You Know . . .”: Ideological Differences in Slippery Slope Thinking

**DOI:** 10.1177/01461672251391893

**Published:** 2025-11-13

**Authors:** Rajen A. Anderson, Daan Scheepers, Benjamin C. Ruisch

**Affiliations:** 1 University of Leeds, UK; 2 Leiden University, The Netherlands; 3 Utrecht University, The Netherlands; 4 University of Kent, Canterbury, UK

**Keywords:** ideology, slippery slope, intuition, reasoning, decision-making

## Abstract

Slippery slope arguments (SSAs) contend that a small, innocuous change will lead to cascading negative consequences. Although SSAs are common in political discourse, they have received little empirical attention in this context. In 15 studies (including samples from four countries and a study of natural language usage on the social media site Reddit), we examine *who* may be most prone to slippery slope thinking and *why* people in general may engage in such thinking. We consider whether individuals of different political ideologies exhibit different degrees of support for SSAs. We test three competing hypotheses that it is (a) political extremists, (b) political liberals, or (c) political conservatives that more strongly endorse SSAs. We consistently find that conservatives endorse SSAs more due to ideological differences in intuitive thinking. We additionally find evidence of these ideological differences in social media behavior, and that slippery slope thinking has consequences for punitive attitudes.

“If you eat one cookie today, tomorrow you’ll want to eat ten, and before you know it, you’ll have gained 10 pounds.” Likely, you have heard (or perhaps even said) such a statement before. Statements like this are called *slippery slope arguments* (SSAs), an intuitive metaphor for arguments with a characteristic structure. While there is not a “canonical” SSA format or formula, most SSAs share a common basic outline: If *relatively innocuous Action A* occurs, *more negative Consequence C* will occur in the future (often with *intermediate Between Steps B_1_–B_n_*); therefore, to prevent the harmful occurrence of *C*, we should avoid taking *A* (Lode, 1999; Schauer, 1985). By connecting a small, seemingly inoffensive change with a larger, worse outcome in the future, SSAs seek to make that initial change itself seem dangerous and thus something to avoid. SSAs of this form are used widely to argue for particular positions, decisions, or policies in domains such as politics (van der Burg, 1991), law (Lode, 1999), and ethics (Lamb, 1988; Launis, 2002).

Traditionally, slippery slope argumentation as a topic has been discussed in philosophy and law (for example, see Volokh, 2003; Walton, 2015). Despite their broad application, however, there have been only a few lines of empirical research into the psychological factors that drive slippery slope thinking. Corner and colleagues (2011) found that people’s endorsement of individual SSAs was based on features of the arguments themselves such as the evaluated probability of the steps in the SSA chain and the degree of category overlap between the initial action and the final consequence. Haigh et al. (2016) found that evaluations of SSAs can also be influenced by source characteristics (i.e., attributes of the person making the argument): For example, SSAs are rated as more persuasive if the conclusion seems consistent with the speaker’s values. Finally, Adelman and colleagues (2021) identified individual differences in general propensities to endorse slippery slope thinking, and that this tendency negatively correlated with factors such as generalized trust in others and sense of control and positively correlated with conspiratorial thinking. In the present research, we extend this past work to better understand the psychology of slippery slope thinking and logical reasoning more broadly.

We center this investigation on the role of political ideologies in slippery slope thinking. Politics is among the real-world domains in which slippery slope thinking is most commonly observed (Stenvoll, 2008): Politicians and pundits frequently employ SSAs to persuade others of certain policy positions (Stenvoll, 2008). Will raising taxes now open the door to greater tax increases downstream? Will increasing military involvement in this conflict lead to participating in other, larger conflicts? Understanding the ideological correlates of slippery slope thinking—who is most persuaded by such arguments and why—will thus provide important practical and theoretical insights into political behavior.

Past research on political psychology appears to support three alternative—and partially conflicting—hypotheses regarding how slippery slope thinking may relate to political ideology. Specifically, different political attitudes— ideological extremity, political liberalism, and political conservatism—have all been shown to relate to distinct psychological correlates that could theoretically predict slippery slope thinking.

## H1: Extremity and Slippery Slope Thinking

Some research suggests that ideological extremists (i.e., individuals who are strongly liberal or strongly conservative, relative to moderates) will be most prone to slippery slope thinking. Political extremists—versus moderates—are argued to be more cognitively rigid (Zmigrod et al., 2020), to engage in greater motivated reasoning (Kahan, 2013; Toner et al., 2013), and to view politics in more simplistic terms (Lammers et al., 2017). Insofar as SSAs offer relatively simplistic predictions and resolutions for complex questions (e.g., clear causal chains between causes and effects), extremists might find these arguments particularly compelling.

## H2: Liberalism and Slippery Slope Thinking

A separate body of work suggests that political liberals (also known as left-wing or progressive individuals) would be more susceptible to slippery slope thinking. Support for this hypothesis comes from research on “concept creep” (Haslam, 2016; Haslam et al., 2021), the gradual expansion in the definition and use of certain categories to encompass a wider set of exemplars. For example, Haslam (2016) argues that the concept of “abuse” has expanded beyond requiring direct physical contact to include new subtypes like emotional abuse and neglect. Because the concepts that have “crept” (i.e., expanded) are primarily related to harm (Haslam, 2016), and liberals place greater moral emphasis on harm (e.g., Graham et al., 2009), liberals are argued to be particularly prone to concept creep (Haidt, 2016; Haslam et al., 2020). Furthermore, concept creep and slippery slope thinking appear to share the same cognitive mechanism: A perceived conceptual overlap between different negative acts (Corner et al., 2011). Thus, concept creep and slippery slope thinking appear to both rely on (negative) concepts being grouped together and evaluated as belonging to a shared category—and both may therefore be more common among liberals.

## H3: Conservatism and Slippery Slope Thinking

Finally, yet another body of psychological research provides reason to predict that slippery slope thinking might instead be more common among political conservatives (also known as right-wing individuals). In particular, conservatives tend to be more averse to change and to place greater value on tradition and maintaining the status quo (i.e., existing states of affairs; Jost, 2017; Jost et al., 2003). Moreover, political conservatives have been argued to rely more strongly on intuition and heuristics than political liberals (Deppe et al., 2015; Talhelm et al., 2015): To the extent that SSAs provide plausible sequences of events, conservatives may be particularly susceptible to their intuitive appeal (Svedholm-Häkkinen & Kiikeri, 2022). By extension, prompts to engage in less intuitive thinking may reduce conservative’s use of slippery slope thinking.

### Present Research

Across 15 studies, we tested these competing hypotheses using correlational, experimental, and natural language processing methodologies. We recruited samples from four different nations in four different languages (Studies 1, 3, and 5–8 recruited from the United States in English, Study 2a from the Netherlands in Dutch, Study 2b from Finland in Finnish, and Study 2c from Chile in Spanish). We also document two real-world implications of slippery slope thinking: social media activity (Study 4) and criminal policy support (Study 8). We report two of our studies in the Supplemental Materials, ruling out a potential alternative explanation regarding gender stereotypes (Study S1) and serving as an initial pilot study for our hypothesized mechanism (Study S2). In our survey studies, we ask participants to self-report their political attitudes, which we then use as a continuous measure of political ideology (from liberal/left wing to conservative/right wing) and additionally use to calculate political extremity (from moderate to extremely [liberal/conservative]). We preregistered all studies besides Studies 1a, 1b, 2a, and 3. All preregistration information, materials, data, and analysis scripts are available on the Open Science Framework (OSF) page for this project, https://osf.io/jvb8d/?view_only=c2ca111c9e0e45ab8bf504bc51d63adb.

## Studies 1a–1d

For the first tests of these hypotheses, we assessed judgments of prototypical SSAs. We collected arguments from logic textbooks and philosophy websites (e.g., the Stanford Encyclopedia of Philosophy), avoiding arguments that (explicitly or tacitly) dealt with political/politicized issues (e.g., abortion, same-sex marriage, euthanasia). Studies 1a-1d followed a similar methodology: Participants rated slippery slope-style arguments and reported their political ideology.

### Study 1a Method

#### Participants

We conducted a power analysis for 80% power and an expected effect size of *r* = .2, based on similar effects in the literature (e.g., Ruisch & Stern, 2021). This suggested a target sample size of 191 participants, which we increased to 200. In all future studies, we aimed to recruit sample sizes of at least this size, increasing when possible based on available resources to further maximize power. In Study 1a, we recruited 200 participants (110 men, 89 women, 1 did not report; *M*_age_ = 34.6, *SD*_age_ = 10.4) from Amazon’s Mechanical Turk (for a discussion of this platform as a research tool, see Buhrmester et al., 2011).

#### Procedure

After providing informed consent, participants were told that they would be asked to rate several arguments according to how logical or illogical they perceived them to be. They were also specifically informed that “we are NOT interested in whether you agree with the conclusion of the argument—we simply wish to know the degree to which you see the conclusion as following logically from the argument that is made.”

After receiving these instructions, participants viewed six SSAs (see Table 1), presented on the screen one at a time (α = .71). We developed these arguments based on naturally occurring SSAs collected in logic textbooks and philosophy websites (e.g., the Stanford Encyclopedia of Philosophy). Participants were asked to rate the soundness of each argument on a 9-point scale ranging from “Completely illogical” to “Completely logical,” with the midpoint labeled “Neither illogical nor logical.”

**Table 1.** The Slippery Slope Arguments Used to Measure Slippery Slope Thinking, Based on Which Study They Were First Used.

Study	Slippery slope argument
1a	If we lower the drinking age, next thing we know kids will get to drive at age ten, and vote at fifteen.
1a	Today late for ten minutes, tomorrow late for an hour, and then someday you will simply cease to show up.
1a	If you break your diet and have one cookie tonight, you will just want to eat 10 cookies tomorrow, and before you know it, you will have gained back the 15 pounds you lost.
1a	If you allow the students to redo this test, they are going to want to redo every assignment for the rest of the year.
1a	You can never give anyone a break. If you do, they’ll walk all over you.
1a	If you give in every time your baby cries, he will always pitch a fit to get what he wants, and he will end up in prison because you never set limits.
1c	If Dan spends that extra $5 today, tomorrow he’ll spend an extra $20, and before you know it he’ll be completely broke.
1c	If Jim eats that extra 100 calories today, tomorrow he’ll eat an extra 500, and before you know it he’ll have gained 15 pounds.
1c	If Simon’s parents let him skip washing the dishes today, then by next week he’ll stop taking out the trash as well, and by the end of the month, he’ll have completely stopped cleaning.
1c	If we let Mark show up a minute late to work today, he’ll be 5 min late tomorrow, and before you know it he’ll be the last one to arrive.
1c	If John gets a bad score on this test, he’ll fail the class. If John fails the class, he’s likely to fail out of school completely.
1c	If Bill doesn’t keep his living room tidy, he’s likely to stop caring about the other rooms as well. If he stops caring about the other rooms, before you know it the whole house will collapse.

Following this, participants were asked to provide their age and gender. Finally, they rated their political orientation on a 9-point scale from “Extremely Liberal” to “Extremely Conservative” (*M* = 3.90, *SD* = 2.27), with the midpoint labeled “Moderate” (a commonly used measure of political ideology; Jost, 2006; Linden et al., 2021). Consistent with past work (e.g., Brandt et al., 2015), we calculated ideological extremity in all studies by “folding over” this scale to assess distance from the midpoint of the scale, resulting in a 5-point scale of extremity ranging from 0 (“Moderate”) to 4 (“Extremely [Liberal/Conservative])” (*M* = 2.10, *SD* = 1.39).

### Study 1b Method

#### Participants

Participants were recruited from a large university in the Midwestern United States. Two hundred and eighty-five participants completed the study. Nine of these participants did not provide their political orientation, leaving an analyzable sample of 276 participants (154 women, 120 men, 2 nonbinary; *M*_age_ = 19.91, *SD*_age_ = 4.28).

#### Procedure

This study was part of a larger battery of questions assessing different research questions. Participants first completed several measures unrelated to the present hypotheses. They then rated the same six SSAs from Study 1a (α = .66). Participants then responded to an issue-based measure of political conservatism, asking them to rate their support for U.S. action on six political issues (α = .77): accepting refugees from other countries (reverse-scored); reducing income inequality among citizens (reverse-scored); pursuing aggressive foreign policy; enforcing border security and immigration policies; promoting lesbian, gay, bisexual, transgender, and queer (LGBTQ) rights (reverse-scored); and promoting abortion rights (reverse-scored). Participants also rated their political orientation on the same self-placement measure from Study 1a (*M* = 4.55, *SD* = 1.87), which was used to calculate political extremity (*M* = 1.44, *SD* = 1.28). Finally, they provided demographic information.

### Study 1c Method

#### Participants

We recruited 251 participants from Mechanical Turk based on the power analysis discussed above. No demographic information was collected.

#### Procedure

The study procedure closely mirrored that of Study 1a, but using a new set of six SSAs that followed a consistent, third-person structure: (ambiguously negative) *Action A* leads to (somewhat worse) *Between Step B*, which in turn leads to (considerably worse) *Consequence C* (see Table 1). Participants saw these arguments in randomized order, rating them on the same 9-point scale from Studies 1a and 1b (α = .78). Participants then indicated their political orientation on the same self-reported 9-point scale from Study 1a (*M* = 4.50, *SD* = 2.37), which we used to calculate political extremity (*M* = 1.96, *SD* = 1.41).

### Study 1d Method

#### Participants

Based on the power analysis outlined above, we set a target sample size of 250 participants, whom we recruited from Mechanical Turk. We received 251 responses (134 women, 117 men; *M*_age_ = 37.89, *SD*_age_ = 11.97).

#### Procedure

The procedure of this study closely mirrored that of our previous studies. Participants were asked to rate the six SSAs first used in Study 1a. In this study, however, rather than being asked to rate how logical these statements were, participants were instead asked to estimate the objective probability that these chains of negative events would actually occur (“In your opinion, what is the objective likelihood that this chain of events would actually occur?”), on a 100-point scale from “Extremely unlikely” to “Extremely likely” with the midpoint labeled “Neither unlikely nor likely” (α = .78). This allowed us to further ensure that the effects we had documented were not driven by any particular feature of the response scale that we used in our previous studies (e.g., differing interpretations of what it means for a statement to be “completely logical”) and allowed us to more directly assess people’s predictions about what is likely to happen given a particular action. Participants then reported political ideology using the 9-point scale from Study 1a (*M* = 4.57, *SD* = 2.31), which we also used to calculate political extremity (*M* = 1.91, *SD* = 1.36).

### Results

We first tested the *extremity and slippery slope* hypothesis (see Table 2 for a summary of results for Studies 1a–1d). We found no consistent evidence of an association between ideological extremity and slippery slope thinking: More extreme versus moderate individuals did not reliably differ in their degree of slippery slope thinking.

**Table 2.** Summary of Linear Regression Analyses for Studies 1a–1d.

Study	Test of liberalism versus conservatism	Test of political extremity
Study 1a	β = .31, *t*(198) = 4.53, *p* < .001	β = -.12, *t*(198) = -1.66, *p* = .098
Study 1b	β = .16, *t*(274) = 2.72, *p* = .007	β = -.07, *t*(274) = -1.14, *p* = .25
Study 1c	β = .30, *t*(249) = 4.92, *p* < .001	β = -.02, *t*(249) = -0.38, *p* = .71
Study 1d	β = .16, *t*(249) = 2.61, *p* = .01	β = -.02, *t*(249) = -0.36, *p* = .72

*Note.* For the test of liberalism versus conservatism, ideology was arranged such that higher values corresponded with more conservative identification. Therefore, the significant positive β weights indicate that conservatism (and not liberalism) was associated with greater slippery slope thinking.

We then turned to testing the *conservatism* and *liberalism* hypotheses. In Studies 1a–1d, we found a significant association between our self-identification ideology measure and ratings of the arguments, indicating a connection between political ideology and slippery slope thinking. The direction of this association was such that in each of the four studies, more conservative (vs. liberal) individuals rated the arguments as being significantly more logical, supporting the *conservatism and slippery slope* hypothesis (see Table 2). Moreover, we conceptually replicated this significant association using the issue-based measure of ideology in Study 1b, β = .20, *t*(274) = 3.29, *p* = .001. The association was also robust to controlling for participant age and gender: Study 1a: β = .31, *t*(195) = 4.54, *p* < .001; Study 1d: β = .18, *t*(247) = 2.90, *p* = .004. Finally, we additionally found that the effect of conservatism on slippery slope thinking was largely the same when examining individual arguments (see SM for more details), suggesting that the association between conservatism and slippery slope thinking manifests to an equivalent degree across different kinds of arguments, contexts, and content.

Taken together, the results from Studies 1a–1d provided clear support for the *conservatism and slippery slope* hypothesis: Participants who were politically conservative tended to evaluate SSAs as more logical. In contrast, there was no support for either the liberalism or the extremity hypotheses in any of these studies. We conducted a supplemental study using women as the agents in the arguments to rule out a potential alternative explanation rooted in gender stereotypes of men being seen as more impulsive and erratic in their behavior (see Study S1 in the SM).

## Studies 2a–2c

We next examined the generalizability of these findings by recruiting samples from beyond the U.S. sociopolitical context: the Netherlands (Study 2a), Finland (Study 2b), and Chile (Study 2c). Each of these countries offers a unique comparison against the United States. The Netherlands has a multi-party (vs. two-party) political system and substantially less political polarization (Adams et al., 2012; Reiljan, 2020). The majority language in Finland, Finnish (*Suomi*), is a Uralic language that differs markedly in its structure from the Indo-European languages (including English and Dutch) that are spoken throughout Europe and the Americas and by a plurality of people worldwide. Given that the “slippery slope” metaphor is linguistic in nature, examining the association between political beliefs and slippery slope thinking in a different linguistic tradition would provide additional generalizability. Finally, Chile differs from these WEIRD countries (Henrich et al., 2010) on key economic and cultural dimensions (Cordano et al., 2010; Guesalaga & Pitta, 2014), while also exhibiting a substantially different sociopolitical trajectory: Unlike the other nations that we examined, which were undergoing either a rightward shift (i.e., The United States under Donald Trump) or remaining relatively politically stable (i.e., Finland and the Netherlands), Chile was in the midst of a substantial leftward shift in its national politics (*el Estallido Social*) that culminated in a rewriting of the nation’s constitution led by progressive voices. Taken together, this cultural, political, and linguistic diversity offered an especially stringent test of the robustness and generalizability of the conservatism-slippery slope relation.

### Study 2a Method

#### Participants

We recruited 214 participants from Prolific Academic (120 men, 92 women, 2 different; *M*_age_ = 28.09, *SD*_age_ = 9.06). All individuals over the age of 18 residing in the Netherlands were eligible to participate. Three participants failed the included attention check, and another 36 participants did not provide a response to the attention check. For consistency with our previous studies, we nonetheless included these participants in the analyses. However, our effects are not substantively altered if these participants are excluded from analyses.

#### Procedure

This study was a conceptual replication of Study 1c. All study materials were translated into Dutch. Participants first provided demographic information, completed the attention check, and answered two questions unrelated to the current hypotheses. They then rated the six SSAs from Study 1c using an 11-point scale ranging from “completely illogical” to “completely logical.” Due to a typing error, one argument was rendered largely nonsensical.^1^ We thus excluded ratings of this argument from analyses, focusing on the other five items (α = .63). However, none of our conclusions are substantively altered if the item was included. Participants then indicated their degree of general political conservatism/liberalism on a 7-point scale from “very left-wing” to “very right-wing” (*M* = 3.47, *SD* = 1.44). Because the term “liberal” has a different meaning in the Netherlands and much of Western Europe, we instead used the more directly analogous terms “right-wing” and “left-wing.” We calculated political extremity in the same manner as in previous studies (*M* = 1.33, *SD* = 0.77).

### Study 2b Method

#### Participants

We recruited 200 participants from Prolific Academic (Palan & Schitter, 2018). All individuals over the age of 18 residing in Finland were eligible to participate. We did not collect demographic information regarding gender or age. Only one participant failed our attention check. For consistency with our previous studies, we nonetheless included this participant in our analyses. Our effects are not substantively altered if this participant is excluded.

#### Procedure

This study was a near-direct replication of Study 2a. All study materials were translated into Finnish. Participants first rated the perceived logic of the six SSAs from Study 2 on the same nine-point scale from our previous studies (α = .78). They also responded to two exploratory questions unrelated to the present hypotheses. Participants then indicated their degree of general political conservatism/liberalism on a 9-point scale from “very liberal” to “very conservative” (*M* = 3.69, *SD* = 1.65), which we also used to calculate political extremity (*M* = 1.75, *SD* = 1.78).

### Study 2c Method

#### Participants

We recruited 200 participants from Prolific Academic. All individuals over the age of 18 residing in Chile were eligible to participate. We did not measure demographic information or include any attention checks.

#### Procedure

This study was a near-direct replication of Study 2a, with all study materials translated into Spanish. Participants first rated the perceived logic of the six SSAs from Study 2a on the same 9-point scale from our previous studies (α = .67). They also responded to two exploratory questions unrelated to the present hypotheses. Participants then indicated their degree of general political conservatism/liberalism on a 9-point scale from “very left/progressive” to “very right/conservative” (*M* = 3.76, *SD* = 1.70), which we also used to calculate political extremity (*M* = 1.70, *SD* = 1.24). Similar to Study 2a, the term “liberal” has a different meaning in Chile than it does in the United States, so we instead used the more directly analogous terms of “left/progressive” and “right/conservative.”

### Results

Conceptually replicating our previous studies, we found a significant association between conservative ideology and ratings of the SSAs, such that more conservative individuals exhibited greater slippery slope thinking (see Table 3). In contrast, we found mixed/weak evidence supporting a link between political extremity and slippery slope thinking (see Table 3). Given the lack of consistent results supporting the *extremity* hypothesis, we no longer examine its effect in our remaining studies (we note that there was also no strong evidence for this hypothesis in section “Internal Meta-Analyses”). Together, the results of Study 2 provided additional convergent evidence for the hypothesized association between conservative ideology and slippery slope thinking by extending this investigation to diverse cultural contexts: the Netherlands, Finland, and Chile. Although these results cannot decisively speak to the degree to which ideological differences in slippery slope thinking generalize to other cultures writ large, they nonetheless demonstrate that these effects are not limited to the U.S. sociopolitical, linguistic, and cultural context and may perhaps reflect more general cognitive differences between more politically liberal and more politically conservative individuals (e.g., Chirumbolo & Leone, 2008; Hibbing et al., 2014; Inbar et al., 2009; Jost et al., 2003).

**Table 3.** Summary of Linear Regression Analyses for Studies 2a–2c.

	Test of liberalism versus conservatism	Test of political extremity
Study 2a (the Netherlands)	β = .19, *t*(212) = 2.80, *p* = .006	β = -.13, *t*(212) = 1.96, *p* = .051
Study 2b (Finland)	β = .22, *t*(198) = 3.15, *p* = .002	β = -.15, *t*(198) = 1.59, *p* = .11
Study 2c (Chile)	β = .24, *t*(198) = 3.41, *p* = .001	β = -.15, *t*(198) = 2.10, *p* = .04

*Note.* For the test of liberalism versus conservatism, ideology was arranged such that higher values corresponded with more conservative identification. Therefore, the significant positive β weights indicate that conservatism (and not liberalism) was associated with greater slippery slope thinking.

## Study 3

The results of our previous studies provided consistent support for an association between conservatism and slippery slope thinking. However, because our previous studies did not include control statements, it is possible that these effects were explained by other factors, such as the specific response scale that we used—e.g., with liberals and conservatives perhaps having different interpretations of what it means for a statement to be “completely logical,” or conservatives simply having a response bias toward the upper end of the scale (and would therefore rate *all* statements as more logical). To assess this possibility, in Study 3, we randomized whether participants were assigned to rate the same SSAs from Studies 1a and 1b or to rate control statements that were similar in content but which did not involve a cascading chain of negative events. We predicted that conservatism would predict endorsement of the SSAs but not the control statements.

### Method

#### Participants

We recruited 400 participants from Mechanical Turk (200 men, 198 women, 1 other, 1 did not respond; *M*_age_ = 38.06, *SD*_age_ = 12.55).

#### Procedure

The procedure of this study closely mirrored that of our previous studies. Participants read the same instructions and then were randomly assigned to either the *slippery slope* condition or the *control* condition. In the *slippery slope* condition, participants rated the same six statements used in Studies 1a and 1b (α = .68; e.g., “If you break your diet and have one cookie tonight, you will want to eat 10 cookies tomorrow, and before you know it, you will have gained back the 15 pounds you lost”). In the *control* condition, participants rated equivalent statements that did not feature a slippery slope structure but mirrored the SSAs in content (α = .68; e.g., “If you break your diet and have one cookie tonight, you’ll have eaten extra calories”). Participants then provided their age, gender, and political orientation using the 9-point scale used in Study 1a (*M* = 4.37, *SD* = 2.49).

### Results and Discussion

We first tested whether the relation between ideology and ratings of the statements differed as a function of condition. As can be seen in Figure 1, we found a marginally significant interaction between ideology and condition, β = .05, *t*(396) = 1.89, *p* = .06. Although not meeting traditional levels of significance, this interaction was consistent with our predictions regarding when ideology would predict endorsement of different arguments. That is, decomposing this interaction revealed a significant association between ideology and ratings of the SSAs, β = .20, *t*(187) = 2.74, *p* = .007, replicating our previous studies. However, there was no significant relation between ideology and ratings of the control statements, β = .04, *t*(209) = 0.63, *p* = .53.

**Figure 1.**
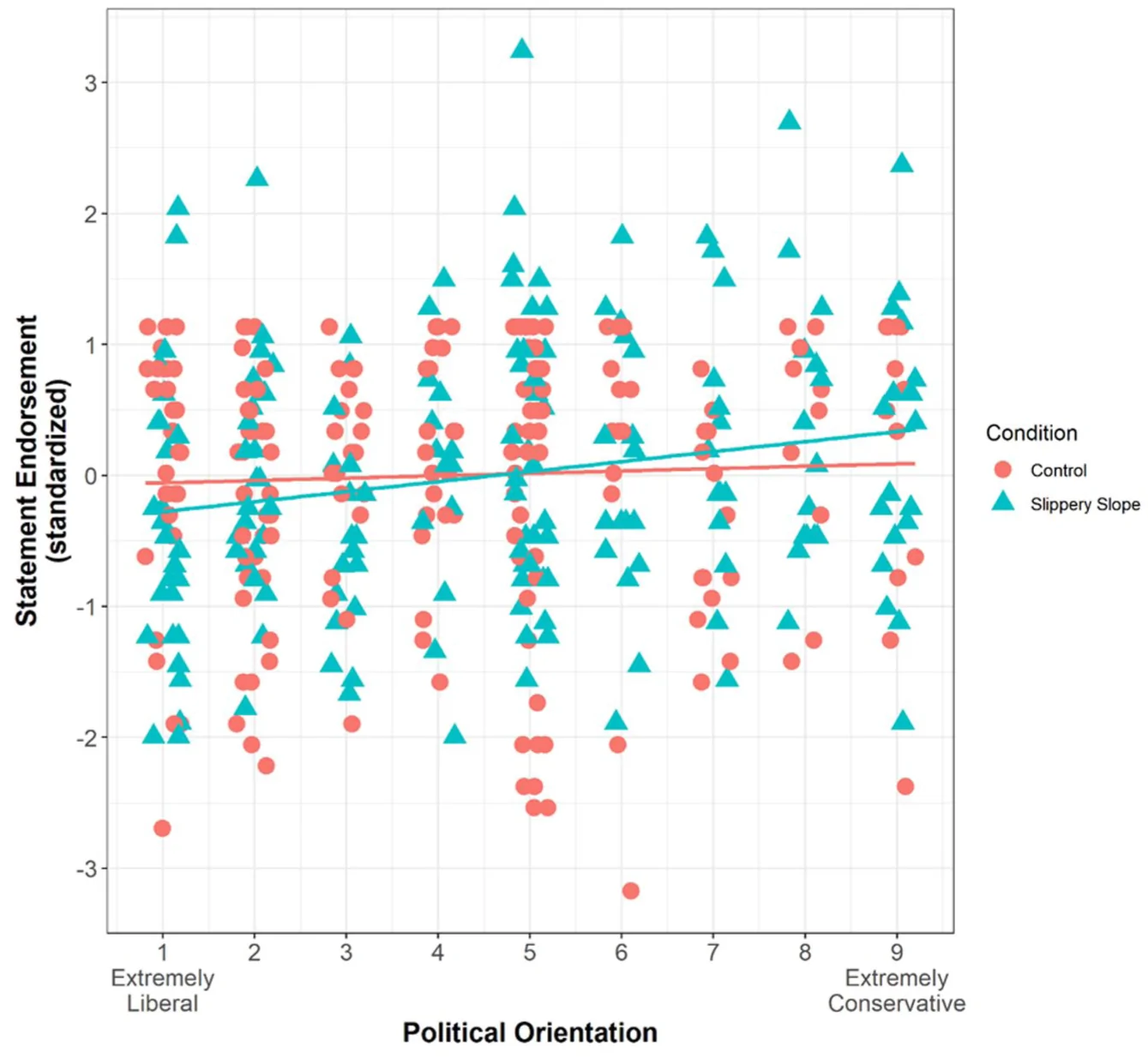
Scatterplot Illustrating the Relation Between Political Orientation and Ratings of the Statements in Study 3. *Note.* Participants who rated slippery slope statements are represented by green triangles. Participants who rated control statements are represented by red circles. Regression lines illustrate the relation between ideology and endorsement of the slippery slope statements (in green) and between ideology and ratings of the control statements (in red).

The results of Study 3 thus provide additional support for the hypothesized association between conservative ideology and slippery slope thinking, while ruling out critical possible alternative explanations. As predicted, we found that the association between conservatism and ratings of these statements did not extend to control statements that did not depict a chain of negative events; rather, conservatives specifically expressed greater endorsement of SSAs.

## Study 4

In Study 4, we employed a natural language processing approach to examine whether the ideological differences we found using the survey methods of Studies 1–3 would replicate in liberals’ and conservatives’ real-world language and communication. We “scraped” comments and posts from the social media website Reddit.com, focusing on four of the larger communities dedicated to discussion of liberal and conservative topics and used ChatGPT to determine the degree to which each comment exhibited slippery slope thinking.

### Method

#### Data Collection

To collect our data, we employed the “Reddit Scraper” plugin for Apify.com, a web-based platform for coding tools, which allows users to extract posts and comments from specific Reddit communities. We collected data in February 2024 and scraped recent posts and comments from r/Democrats (13,943 unique comments), r/Liberal (17,226 unique comments), r/Republican (10,527 unique comments), and r/Conservative (15,760 unique comments). This yielded ~31k comments from politically liberal communities (i.e., r/Democrats and r/Liberal) and ~26k comments from politically conservative communities (i.e., r/Republican and r/Conservative). In addition to the text of the comments, we also collected information on the number of “upvotes” for each comment given by other Reddit users as a measure of general approval toward the comment.

#### Slippery Slope Coding

After collecting the data, we coded the data for the degree to which each comment and post exhibited slippery slope thinking. Given the amount of text data, we turned to ChatGPT for assistance with this task. Research has found that ChatGPT can code large amounts of text information based on psychologically relevant dimensions (Rathje et al., 2023). Using the “rgpt3” package for R, we queried ChatGPT using the following prompt: “How much does the following text rely on slippery slope thinking? Answer using only a number: 1 = not at all, 2 = some, 3 = a lot.” To improve clarity of interpretation, we recoded ChatGPT’s codes by shifting values down by 1, such that 0 = not at all, 1 = some, 2 = a lot.

Following recommendations on using coding algorithms for such tasks (Thelwell, 2018) and to ensure that ChatGPT was coding text in the intended manner, the first and third authors of the present paper manually coded 409 comments (~102 comments per Reddit community) using the same criteria provided to ChatGPT and without seeing which community each comment was from nor ChatGPT’s code for the comment. By technical error, ChatGPT failed to code 18 of these comments, leaving 391 comments to compare between the human codes and the ChatGPT codes. There was exact agreement between the human codes and the ChatGPT codes for 67.5% of the comments, with Cronbach’s α = .631, Cohen’s *κ* = .34, and intraclass correlation coefficient (ICC) = .461. Given this relatively high agreement rate and the relative complexity of the coding task, we proceeded with using ChatGPT’s codes for the full sample of comments.

### Results

Supporting our prediction and consistent with our previous studies, we found that comments made to politically right-leaning Reddit communities (i.e., r/Conservative and r/Republican; *M* = 0.47, *SE* = 0.005) exhibited greater slippery slope thinking than comments made to left-leaning Reddit communities (i.e., r/Democrats and r/Liberal; *M* = 0.40, *SE* = 0.004), *t*(51147) = 10.80, *p* < .001, *d* = 0.10. This effect of ideology holds when controlling for the number of words in the comment, *p* < .001. Although the effect size was relatively small, these results suggest that political ideology influences slippery slope thinking not just in responses to arguments (as seen in Studies 1–3) but also in the actual arguments and language employed by people of different political leanings.

As an exploratory analysis, we also examined whether the degree of slippery slope thinking exhibited by a comment predicted the number of “upvotes” it received—that is, do other users approve more of “slippery slope” style comments? To do so, we tested a linear regression model predicting the number of upvotes each comment received, based on the slippery slope rating of the comment and controlling for the political leaning of the Reddit community and the number of words in the comment. We found a significant effect of the slippery slope rating: comments that exhibited greater slippery slope thinking received a greater number of upvotes, *b* = 2.81, *SE* = 0.34, *t* = 8.21, *p* < .001. Given the design of Reddit and similar social media platforms, comments that received more upvotes are more likely to be seen by other users (Proferes et al., 2021). In addition, social media behavior—even small trends—can have larger real-world impacts when scaled up, considering the millions of users on many platforms (e.g., Brady et al., 2023; Mooijman et al., 2018). Thus, these results highlight the potential real-world impact of slippery slope thinking—and the observed ideological differences in the propensity to engage in that kind of thinking.

## Study 5

In Study 5, we wished to provide an even more stringent test of our hypotheses to more decisively determine whether these effects represent ideological differences *specifically* in slippery slope thinking—and not, for example, ideological differences in broader traits such as general pessimism or negativity bias (e.g., Amodio et al., 2007; Hibbing et al., 2014; Shook & Fazio, 2009; for alternative accounts, see Choma et al., 2014; Choma & Hodson, 2017; Fiagbenu et al., 2021), or other personality-type traits that have been shown to differ between liberals and conservatives, such as belief in a just world (Dittmar & Dickinson, 1993; Hafer & Choma, 2009).

Previous research on ideological differences in just world beliefs shows that conservatives tend to view the world as being a more just place (Dittmar & Dickinson, 1993), where people receive consequences that are befitting of their actions. From this perspective, it may be that conservatives rate SSAs as more logical because they believe that a person who engages in an ambiguously bad action (e.g., breaking one’s diet, not tidying one’s living room) deserves—and thus will receive—negative consequences (e.g., gaining weight, having the house collapse). Furthermore, conservatives may simply exhibit greater pessimism or negativity—if a negative action occurs, the expectation may be for conservatives that more bad actions will follow. Alternatively, if, as we predict, these effects specifically relate to slippery slope thinking, they should emerge only for arguments depicting a slippery slope pattern—i.e., those that depict an ambiguously negative action leading, via a series of (relatively plausible) intervening steps, to a substantially worse outcome.

To test this question, we contrasted ratings of SSAs with ratings of statements depicting matched action-outcome pairs. Specifically, as in our previous studies, participants in the slippery slope condition viewed SSAs of the prototypical form: (ambiguously negative) *Action A* leads to (somewhat worse) *Between Step B*, which in turn leads to (considerably worse) *Consequence C*. Participants in the “skipped step” condition viewed identical statements but without the intervening step—advancing directly from *A* to *C*. Consistent with our proposed account, we predicted that conservatives would rate arguments as more logical *only* when a plausible (potentially intuitively appealing) causal pathway—*Between Step B*—was provided between *Action A* and *Consequence C*. As noted above, part of the psychological appeal of SSAs is the perceived conceptual similarity between the *A* and *C* of the argument (Corner et al., 2011). By removing *B*, the link between *A* and *C* may seem less justified to individuals reviewing the argument. In the absence of this intervening step, ideological differences should be attenuated.

### Method

#### Participants

We recruited 500 participants from Mechanical Turk (269 women, 230 men, 1 nonbinary; *M*_age_ = 37.45, *SD*_age_ = 12.47^2^).

#### Procedure

The procedure of this study closely mirrored those of our previous studies, with one exception. Participants were randomly assigned to either the *slippery slope* condition or the *skipped step* condition. Participants in the *slippery slope* condition rated the same six vignettes from Study 1c. Participants in the *skipped step* condition rated identical statements in which the intervening step had been removed (e.g., rather than “If Dan spends that extra $5 today, tomorrow he’ll spend an extra $20, and before you know it he’ll be completely broke,” they rated the following statement: “If Dan spends that extra $5 today, he’ll soon be completely broke.”). In both conditions, participants rated how logical the statements were using the same 9-point scale used in previous studies (slippery slope α = .85; skipped step α = .77). After answering the six items, they then provided their political orientation using the 9-point scale of self-reported ideology (*M* = 4.41, *SD* = 2.29) and other demographic information.

### Results

We conducted a multiple linear regression, with political orientation, condition, and the interaction term predicting the mean rated logic of the statements. There was a significant main effect of both political conservatism, β = .09, *t*(495) = 2.15, *p* = .03, and condition, β = .28, *t*(495) = 6.59, *p* < .001. The latter relationship meant that participants rated the statements as more logical in the *slippery slope* condition than in the *skipped step* condition. In addition, as anticipated, we found a significant interaction between political ideology and condition in predicting ratings of the statements, β = .11, *t*(495) = 2.51, *p* = .01 (see Figure 2). The pattern of this interaction was as predicted: Replicating our previous studies, in the slippery slope condition, we found a significant relation between ideology and ratings of the statements, such that conservatives exhibited greater slippery slope thinking than liberals, β = .20, *t*(244) = 3.22, *p* = .001. However, for the skipped step statements, this association was completely eliminated, and there was no significant association between ideology and ratings of the statements, β = −.01, *t*(251) = −0.22, *p* = .83.

**Figure 2.**
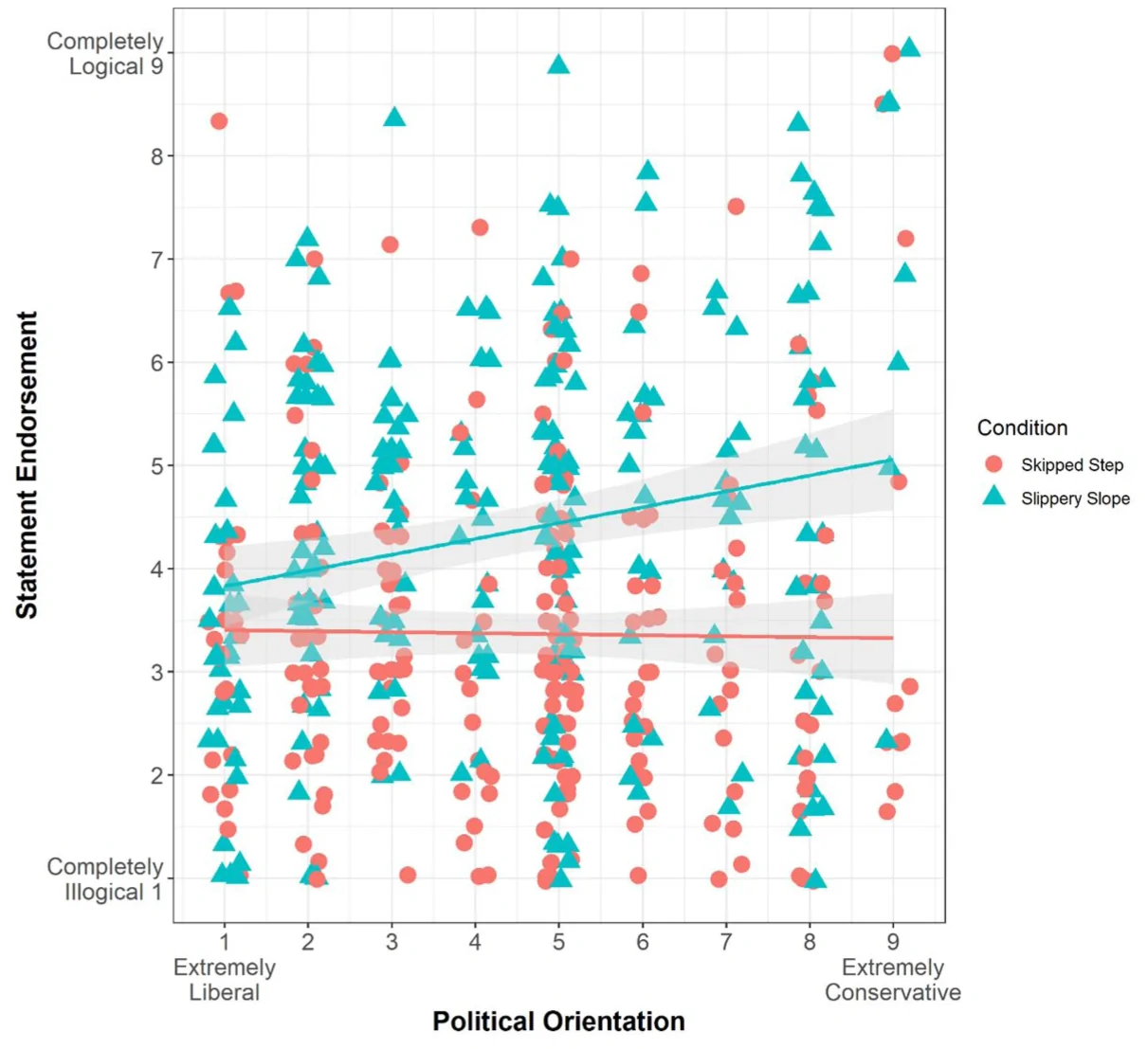
Scatterplot Illustrating the Relation Between Political Orientation and Ratings of the Statements in Study 5 *Note*. Participants who rated slippery slope statements are represented by green triangles. Participants who rated skipped step statements are represented by red circles. Regression lines illustrate the relation between ideology and endorsement of the slippery slope statements (in green) and between ideology and ratings of the skipped step statements (in red). Shaded areas indicate the 95% confidence intervals of the regression lines.

### Discussion

The results of Study 5 provide some initial insight into the mechanisms of the *conservatism and slippery slope* effect and the psychological processes of slippery slope thinking more generally. Specifically, we found that the ideological difference in the perceived logic of slippery slope-like arguments emerges *only* when the arguments exhibited the prototypical structure of *Action A* leads to *Between Step B* which ends with *Consequence C*. When the connecting link of *B* is removed, conservatives no longer differ from liberals in how logical they rate the argument. These results may suggest that part of what may give SSAs their cognitive appeal is how intuitively the various steps flow from each other—when a connection is severed, the “logic” of the motion from *A* to *C* may no longer seem as intuitive or likely to occur (Corner et al., 2011). We more directly examine the role of intuition in slippery slope thinking and the conservatism effect in following studies.

## Study 6

In Studies 6 (correlational) and 7 (experimental), we examined the psychological mechanism underlying the conservatism-slippery slope relation. Extending the results of Study 5, and based on a pilot study (see Study S2 in SM), we focused on the use of intuitive thinking styles. Research has found that liberals and conservatives differ in the degree to which they rely on intuitive/experiential versus deliberative/rational thinking (Deppe et al., 2015; Sterling et al., 2016; Talhelm et al., 2015, although see Kahan, 2013), with conservatives being more likely to “go with their gut” and provide more intuitive responses, and liberals tending to engage in more extensive deliberation. Given that slippery slope argumentation has been argued to be strongest when it is more intuitively appealing (Corner et al., 2011; Svedholm-Häkkinen & Kiikeri, 2022; Walton, 2010), we hypothesized that ideological differences in intuitive thinking might also help to explain these differences in slippery slope thinking.

### Method

#### Participants

Based on a power analysis performed on the effect size from a pilot study (see Study S2 in SM), we set a target sample size of 400 participants. We received 404 complete responses recruited from Mechanical Turk (218 women, 186 men; *M*_age_ = 38.46, *SD*_age_ = 12.16).

#### Procedure

Participants first rated 12 SSAs: the six arguments from Study 1a and the six arguments from Study 1c (α = .92). We measured individual differences in intuitive/deliberative thinking in three ways: (a) Participants completed the CRT-2 (Thomson & Oppenheimer, 2016), which assesses an individual’s tendency to override intuitive, but incorrect, responses to arrive at a correct answer (e.g., “Emily’s father has three daughters. The first two are named April and May. What is the third daughter’s name?”; intuitive answer: June, correct answer: Emily). We summed the number of incorrect responses to create the first measure of intuitive thinking. We also used (b) the Rationality scale (e.g., “I enjoy intellectual challenges.”; α = .92) and the (c) Experientiality scale (e.g., “I like to rely on my intuitive impressions.”; α = .87) from the Rational–Experiential Inventory (Pacini & Epstein, 1999). To form a composite measure of intuitive thinking, we reverse-coded the Rationality scale and then standardized (*z*-scored) and averaged together the three measures to create a single index of intuition versus deliberation, as specified in our preregistration documentation. Finally, participants self-reported their political ideology using the 9-point scale from previous studies (*M* = 4.99, *SD* = 2.51) and reported their age and gender.

### Results

Replicating our previous results, we found a significant association between political ideology and slippery slope thinking, such that more conservative participants rated the arguments as more logical, β = .36, *t*(402) = 7.70, *p* < .001. As predicted, we also found a significant relation between political orientation and our composite measure of intuitive (vs. deliberative) thinking, such that more conservative individuals exhibited greater reliance on intuition, β = .27, *t*(402) = 5.53, *p* < .001. Finally, as predicted, we also found—using PROCESS Model 4 with 10,000 bootstrapped samples (Hayes, 2018)—that intuitive thinking statistically mediated the association between ideology and slippery slope thinking, indirect effect: β = .10, 95% confidence interval (CI) = [.06, .15], providing an initial indication that ideological differences in intuition may explain the conservatism-slippery slope association.

## Study 7

In Study 7, we used an experimental manipulation to induce greater deliberative thinking among participants. If, as hypothesized, endorsement of SSAs is driven by more intuitive versus deliberative thinking, then leading people to engage in more deliberative processing should decrease endorsement of slippery slope thinking. Critically, this manipulation should also attenuate the relation between ideology and slippery slope thinking—specifically, by decreasing conservatives’ endorsement of these arguments (cf. Stern & West, 2016).

### Method

#### Participants

We preregistered a target sample size of 500 participants, whom we recruited from Mechanical Turk. We received 501 complete responses. No demographic information was collected.

#### Procedure

After providing informed consent, participants were randomly assigned to either the *control* condition or the *deliberation* condition. For participants in the *control* condition, the study was a direct replication of Study 1c, rating the six SSAs on the same 9-point scale used in previous studies. Participants in the *deliberation* condition were led to engage in more deliberative (vs. intuitive) thinking. Because past research has found that it is difficult to alter liberals’ and conservatives’ habitual reliance on intuition versus deliberation (e.g., Deppe et al., 2015, who failed to find effects of established priming manipulations across 4 studies), our manipulation of deliberative thinking used a more powerful, multi-pronged approach: Participants in this condition first received instructions encouraging them to take a more deliberative (vs. intuitive) approach, and they were then asked to check a box confirming that they would do so. In addition, the response scale for each SSA did not appear until 10 s had elapsed, further encouraging participants to deliberate carefully before making a judgment. The measure of slippery slope thinking showed good reliability (α = .78), so we averaged the items together to form our index of slippery slope thinking. After completing the slippery slope items, participants indicated their political ideology using the 9-point scale as in previous studies (*M* = 4.43, *SD* = 2.29).

### Results

We first tested whether our manipulation of deliberative thinking influenced participants’ ratings of the SSAs. As predicted, we found that the manipulation had a significant effect on slippery slope thinking, *t*(499) = 3.34, *p* = .001, *d* = .30. Participants who were led to adopt a more deliberative thinking style rated the arguments as significantly less logical (*M* = 4.00, *SD* = 1.54) than did those in the control condition (*M* = 4.49, *SD* = 1.75).

We next tested whether the effect of the manipulation differed between liberals and conservatives. As previously discussed, we predicted that the manipulation would have a stronger effect on conservatives’ ratings of the arguments, since they are higher in intuitive (vs. deliberative) thinking at baseline, but that it would have less of an impact among liberals, who are relatively higher in deliberative (vs. intuitive) thinking at baseline. We conducted a multiple linear regression, with political orientation, condition, and the interaction term predicting slippery slope thinking.

This interaction did not reach the traditional threshold for statistical significance, and thus, caution in interpretation is warranted, β = −.07, *t*(497) = 1.62, *p* = .105. However, the pattern of effects largely supported our preregistered predictions (see Figure 3). Among conservatives (i.e., those above the midpoint of the political ideology scale; *n* = 141), the manipulation had a statistically significant—and rather substantial—effect on argument ratings, *t*(139) = 3.18, *p* = .002, *d* = 0.54. Conservatives in the *deliberation* condition (*M* = 4.00, *SD* = 1.71) exhibited a reduction in endorsement of the SSAs compared to conservatives in the *control* condition (*M* = 4.97, *SD* = 1.92) that corresponded to approximately 1-point on the 9-point scale. Among liberals (i.e., those below the midpoint of the political ideology scale; *n* = 239), conversely, there was no significant effect of the manipulation, *t*(237) = 1.04, *p* = .30, *d* = 0.13—even despite the larger number of liberal participants and thus greater statistical power to detect an effect. The pattern of effects was directionally consistent with that among conservatives, although the effect was much smaller, corresponding to a mean difference of 0.21 on the 9-point scale (only 21% of the effect observed among conservatives).

**Figure 3.**
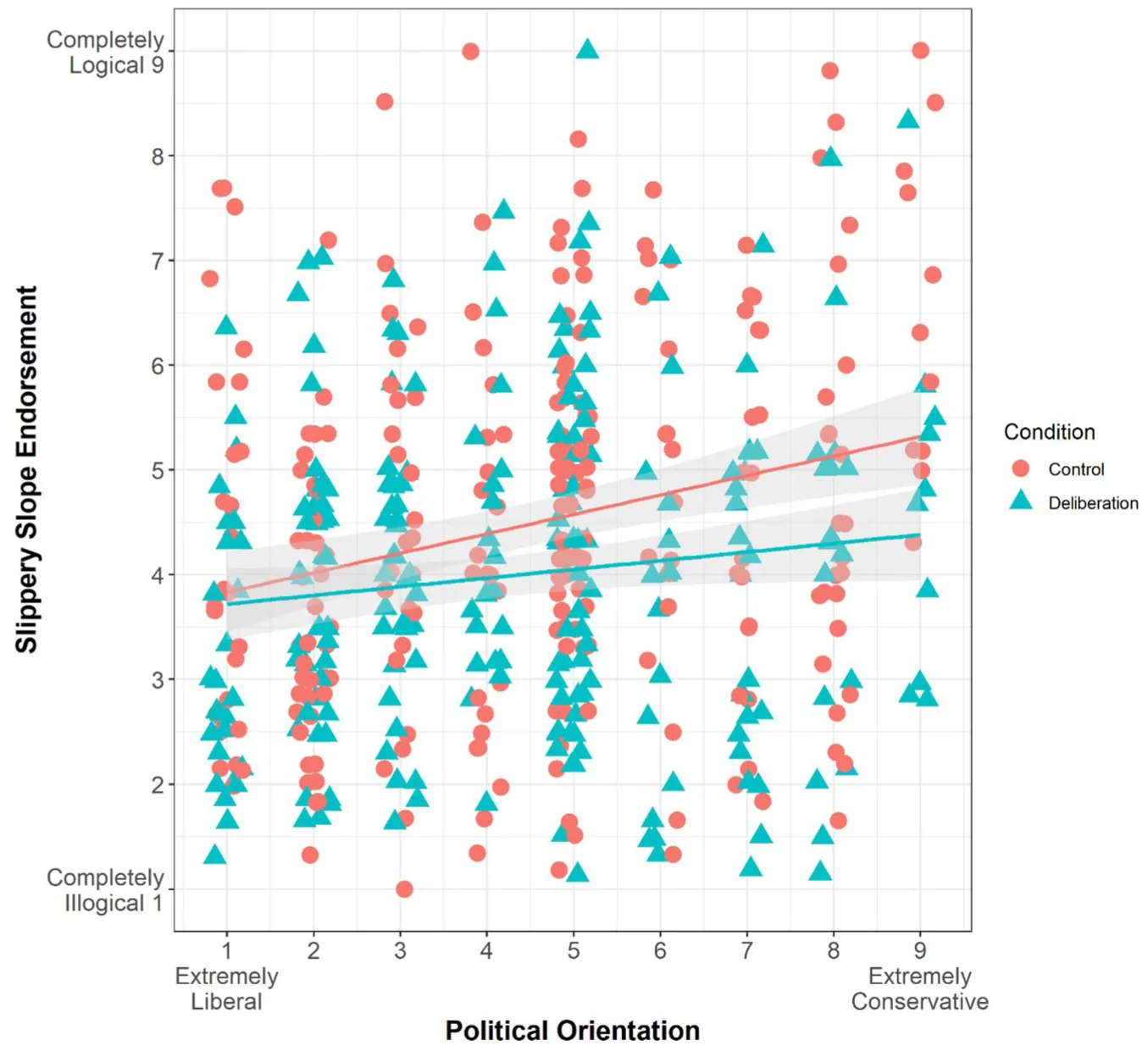
Scatterplot Illustrating the Relation Between Political Orientation and Ratings of the Slippery Slope Arguments in Study 7 *Note.* Participants in the control condition are represented by red circles. Participants in the deliberation condition are represented by green triangles. Regression lines illustrate the relation between ideology and endorsement of the slippery slope statements in the control condition (in red) and deliberation condition (in green). Shaded areas indicate the 95% confidence intervals of the regression lines.

Viewed from another angle, in the control condition, we replicated the previously observed association between ideology and slippery slope thinking, β = .25, *t*(251) = 4.00, *p* < .001, with more conservative participants expressing greater endorsement of the arguments. However, when participants were led to adopt a more deliberative thinking style, these ideological differences were attenuated: The relation between ideology and slippery slope thinking was reduced by half, β = .12, *t*(246) = 1.92, *p* = .06. Notably, the size of this reduction in slopes (β = .12) was comparable to the indirect effect of deliberative thinking we observed in our mediation models in Study 6 (β = .10).

## Study 8

In Study 8, we examined the policy support consequences of slippery slope thinking. We focused on support for harsh criminal sentencing, such as mandatory minimum sentences for habitual offenders (e.g., “three strikes” laws in the United States). We reasoned that individuals who engage in slippery slope thinking—and who should therefore expect that an offender will commit more egregious acts in the future (Anderson et al., 2023)—would exhibit greater support for such policies. As such, ideological differences in slippery slope thinking might partially explain conservatives’ greater support for harsh sentencing (Gerber & Jackson, 2016).

### Method

#### Participants

We preregistered a target sample size of 300 participants, whom we recruited from Prolific. We received 301 complete responses. We excluded one person for failing our attention check item, leaving a final sample of 300 participants (*M_age_* = 42.84, *SD* = 13.99; 137 men, 158 women, 2 “nonbinary,” 1 “questioning,” 1 “prefer not to answer,” 1 blank; 239 White, 17 Black, 31 Asian, 28 Latino/a or Hispanic; median education = 4-year degree; median income = $50,000–$59,999).

#### Procedure

Participants completed a modified version of Study 1c. In addition to rating the six SSAs (α = .80), participants also completed the eight-item Punitive Attitudes Toward Crime scale (Chiricos et al., 2004; α = .92). This scale measures participants’ support for various proposed policies for dealing with crime in the United States. Participants indicated their support on an 11-point scale from “Not at all supportive” to “Very supportive” to policies like “Making sentences more severe for all crimes” and “locking up more juvenile offenders.” Participants were randomized to complete either the SSAs or the Punitive Attitudes Toward Crime scale first. After completing these two scales, participants completed demographic measures (including their political ideology as in Study 1a; *M* = 4.78, *SD* = 2.86) and an attention check item.

### Results

Replicating our previous studies, we again found that political ideology was associated with ratings of the SSAs, with more conservative individuals tending to rate them as more logical than more liberal individuals, β = .24, *t*(298) = 4.22, *p* < .001. This effect of ideology on slippery slope thinking held when controlling for participants’ age, education levels, and income, β = .26, *t*(295) = 4.48, *p* < .001. More conservative individuals also tended to more strongly support harsher criminal sentencing policies, β = .53, *t*(298) = 10.70, *p* < .001. Critically, we also found that slippery slope thinking significantly and positively predicted greater support for harsher criminal sentencing policies, β = .25, *t*(298) = 4.47, *p* < .001. This effect of slippery slope thinking on criminal sentencing support holds when controlling for participants’ age, education levels, and income, β = .24, *t*(295) = 4.42, *p* < .001. In addition, using PROCESS Model 4 (Hayes, 2018), we found that slippery slope thinking statistically mediated the association between political ideology and support for harsh criminal sentencing policies, β = .03, 95% CI (.005, .06). These findings demonstrate the potential real-world implications of slippery slope thinking, suggesting that ideological differences in slippery slope thinking may help explain ideological differences in criminal policy positions.

## Internal Meta-Analyses

Based on best-practice recommendations (Goh et al., 2016; Lakens & Etz, 2017; Mcshane & Böckenholt, 2017), we also conducted two internal meta-analyses of the survey samples (i.e., all studies except Study 4) to increase statistical power and more accurately determine the mean effect size across studies (Braver et al., 2014; Cohn & Becker, 2003). Specifically, we examined the mean effect size of ideology and of political extremity on slippery slope thinking.

As seen in Figure 4, the model for conservatism revealed that the average effect size across these studies was β = .24, *SE* = .02, *z* = 12.97, *p* < .001, 95% CI = [.20, .27]. Cochran’s *Q*-test suggested that there was no significant heterogeneity in the observed effect sizes, *Q*(13) = 15.54, *p* = .27. The model for extremity (see Figure 5) revealed that the average effect size across these studies was β = −.04, *SE* = .03, *z* = 1.54, *p* = .12, 95% CI = [−0.09, 0.01]. Cochran’s *Q*-test suggested that there was significant heterogeneity in the observed effect sizes, *Q*(13) = 36.50, *p* < .001. Specifically, Study 6 had a significant positive association between political extremity and slippery slope thinking. However, given that Study 6 was methodologically very similar to Study S2 (which did not have a significant effect), this is likely a Type I error.

**Figure 4.**
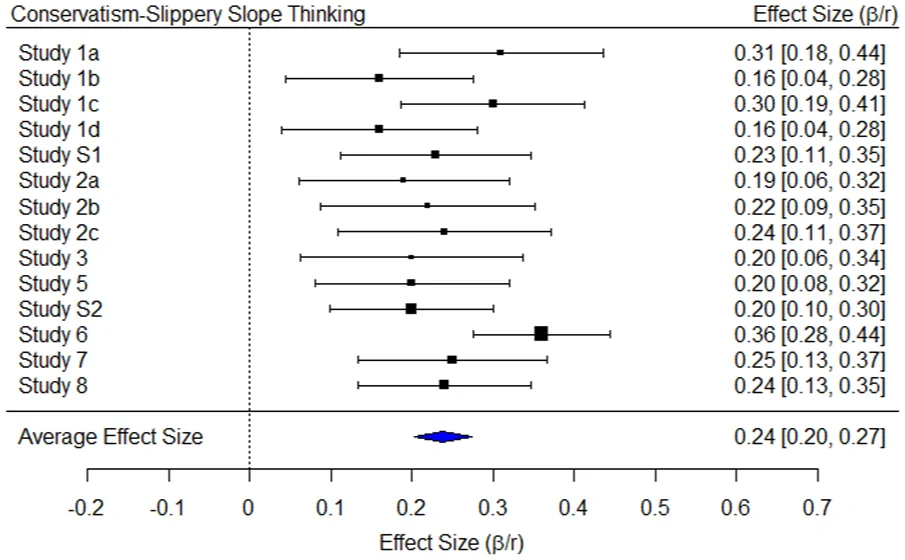
Forest Plot of Effect Sizes of Conservatism–Slippery Slope Thinking Relationship for All Studies (Except Study 4) *Note.* Average effect size (β/*r*) based on a random-effects meta-analysis model.

**Figure 5.**
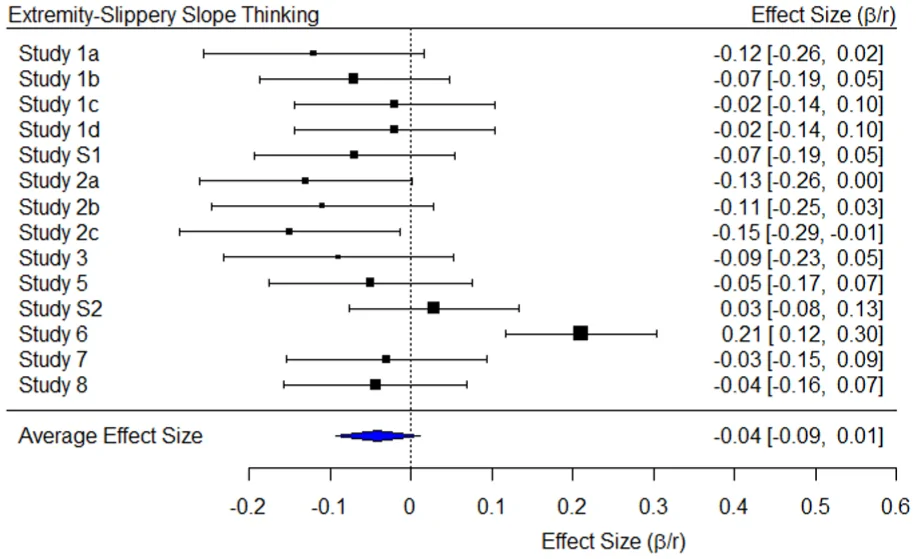
Forest Plot of Effect Sizes of Extremity–Slippery Slope Thinking Relationship for All Studies (Except Study 4) *Note.* Average effect size (β/*r*) based on a random-effects meta-analysis model.

## General Discussion

In 15 studies employing correlational, experimental, and natural language processing designs and with samples recruited from four countries, we tested three competing hypotheses derived from the literature regarding the relation between slippery slope thinking and political beliefs. We found consistent support only for the hypothesis that political conservatism would predict slippery slope thinking: More conservative individuals engaged in slippery slope thinking to a greater degree than more liberal individuals. Furthermore, contrary to one prediction derived from past literature (Fernbach et al., 2013; Zmigrod et al., 2020), we found that ideological extremity did not predict slippery slope thinking. These effects replicated across a variety of cultural, political, and linguistic contexts (Study 2). As boundary conditions, we found that changing the structure of the specific arguments weakened the association between conservatism and endorsement of the arguments (Studies 3 and 5). Moreover, we found that conservatives’ greater slippery slope thinking is at least partially driven by their greater use of intuitive (vs. deliberative) processing (Studies 6 and 7). Importantly, this tendency to engage in slippery slope thinking can have downstream consequences, including in the language used on social media (Study 4) and in criminal policy support (Study 8).

Our work connects to broader themes in political psychology regarding whether and how individual differences in cognitive tendencies relate to ideological beliefs. For example, research has found that liberals and conservatives differ in their propensity toward analytical versus intuitive thinking styles (Talhelm et al., 2015) and their degree of judgment confidence (Ruisch & Stern, 2021). Our studies identify an important new domain of ideological asymmetries, documenting differences in how liberals and conservatives evaluate arguments and make predictions of potentially negative events. Our results suggest that conservatives and liberals differ not just in *what* they believe (i.e., the content of their belief systems) but also in *how* they evaluate the likelihood of different future events given the same set of starting conditions. The greater tendency among conservatives to engage in slippery slope thinking may lead to a “catastrophizing” mind-set, whereby small societal changes seem more disastrous than they are.

Although our findings documented no evidence linking ideological extremity and domain-general slippery slope thinking, it may still be possible that ideological extremists may be more susceptible to slippery slope thinking under particular circumstances. For example, political extremism may predict slippery slope thinking in the political domain, particularly when a given SSA aligns with preferred political positions. Such a pattern of effects would be consistent with research showing that strongly committed partisans exhibit greater motivated reasoning in support of favored conclusions (Ditto et al., 2019; Gampa et al., 2019; Kahan, 2013). Future work might directly examine the relation between political extremity and slippery slope thinking in political contexts to shed light on these questions.

To our knowledge, this research represents one of the first systematic empirical examinations of individual differences in the tendency toward slippery slope thinking and why some people are more likely to endorse SSAs (i.e., intuitive vs. deliberative thinking). As a limitation, we acknowledge that several of our studies had a relatively liberal skew in their samples (as indicated by mean political ideology being on the liberal side of the scale). This suggests that, with more politically balanced samples (i.e., relatively more conservative participants), the association between conservatism and slippery slope thinking may be even *stronger* than what we observed. Future work may also attempt to experimentally manipulate slippery slope thinking when considering its downstream consequences: For example, if people are primed to think that immoral behavior tends to follow a slippery slope pattern (as actually suggested by some past research, e.g., Garrett et al., 2016; Welsh et al., 2015), does that increase their punishment for wrongdoers? Our findings can also offer new predictions about what other psychological traits and motivations are likely to correlate with slippery slope thinking. For example, one line of past research by Adelman et al. (2021) found that a person’s tendency to engage in slippery slope thinking was correlated with belief in conspiracy theories. Given that conspiratorial thinking, too, is driven in part by the use of intuition (Swami et al., 2014), our findings suggest that intuitive thinking is likely to provide a connection between slippery slope argumentation and conspiratorial thinking as well.

To conclude, we tested competing predictions regarding how ideological beliefs would relate to an understudied domain of reasoning and argumentation. We consistently found that political conservatives (vs. liberals) tend to engage in greater slippery slope thinking, an effect explained by differences in intuitive thinking. Given the widespread use of SSAs in politics and other domains (Stenvoll, 2008), we anticipate that our results can help explain and contextualize other liberal-conservative asymmetries in cognition and behavior, including in communication styles and policy support. Given the relative lack of empirical research on the topic, we believe that our findings offer a foundation for understanding slippery slope thinking and will prove generative for future research and theory.

## Supplemental Material

sj-docx-1-psp-10.1177_01461672251391893 – Supplemental material for “And the Next Thing You Know . . .”: Ideological Differences in Slippery Slope Thinking Supplemental material, sj-docx-1-psp-10.1177_01461672251391893 for “And the Next Thing You Know . . .”: Ideological Differences in Slippery Slope Thinking by Rajen A. Anderson, Daan Scheepers and Benjamin C. Ruisch in Personality and Social Psychology Bulletin

## Data Availability

All data, syntax, materials, and preregistration documentation can be found on the Open Science Framework (OSF) at https://osf.io/jvb8d/?view_only=c2ca111c9e0e45ab8bf504bc51d63adb.

## Notes

1. For the item concerning the consequences of failing to tidy one’s house, the word “*huid*” (skin) was inadvertently entered in place of “*huis*” (house), thereby effectively contending that failing to organize one’s living room would lead to bad skin.

2. One participant provided the year they were born (“1991”) rather than their age. The age for this participant was recoded as “29,” their most likely age at the time of the study.

## References

[bibr1-01461672251391893] Adams J. De Vries C. E. Leiter D. (2012). Subconstituency reactions to elite depolarization in the Netherlands: An analysis of the Dutch public’s policy beliefs and partisan loyalties, 1986–98. British Journal of Political Science, 42(1), 81–105. 10.1017/S0007123411000214

[bibr2-01461672251391893] Adelman L. Verkuyten M. Cárdenas D. Yogeeswaran K. (2021). On a slippery slope to intolerance: Individual difference in slippery slope beliefs predict outgroup negativity. Journal of Research in Personality, 94, 104141. https://doi.org/10.1016/j.jrp.2021.104141

[bibr3-01461672251391893] Amodio D. M. Jost J. T. Master S. L. Yee C. M. (2007). Neurocognitive correlates of liberalism and conservatism. Nature Neuroscience, 10(10), 1246–1247. 10.1038/nn1979 17828253

[bibr4-01461672251391893] Anderson R. A. Ruisch B. C. Pizarro D. A. (2023). On the highway to hell: Slippery slope perceptions in judgments of moral character. Personality and Social Psychology Bulletin, 50(5), 679–693. 10.1177/01461672221143022 36602035

[bibr5-01461672251391893] Brady W. J. McLoughlin K. L. Torres M. P. Luo K. F. Gendron M. Crockett M. J. (2023). Overperception of moral outrage in online social networks inflates beliefs about intergroup hostility. Nature Human Behaviour, 7(6), 917–927. 10.1038/s41562-023-01582-0 37037990

[bibr6-01461672251391893] Brandt M. J. Evans A. M. Crawford J. T. (2015). The unthinking or confident extremist? Political extremists are more likely than moderates to reject experimenter-generated anchors. Psychological Science, 26(2), 189–202. https://doi.org/10.1177/0956797614559730 10.1177/0956797614559730 25512050

[bibr7-01461672251391893] Braver S. L. Thoemmes F. J. Rosenthal R. (2014). Continuously cumulating meta-analysis and replicability. Perspectives on Psychological Science, 9(3), 333–342. 10.1177/1745691614529796 26173268

[bibr8-01461672251391893] Buhrmester M. Kwang T. Gosling S. D. (2011). Amazon’s Mechanical Turk: A new source of inexpensive, yet high-quality, data? Perspectives on Psychological Science, 6(1), 3–5. 10.1177/1745691610393980 26162106

[bibr9-01461672251391893] Chiricos T. Welch K. Gertz M. (2004). Racial typification of crime and support for punitive measures. Criminology, 42(2), 358–390. 10.1111/j.1745-9125.2004.tb00523.x

[bibr10-01461672251391893] Chirumbolo A. Leone L. (2008). Individual differences in need for closure and voting behaviour. Personality and Individual Differences, 44(5), 1279–1288. 10.1016/j.paid.2007.11.012

[bibr11-01461672251391893] Choma B. L. Hanoch Y. Hodson G. Gummerum M. (2014). Risk propensity among liberals and conservatives: The effect of risk perception, expected benefits, and risk domain. Social Psychological and Personality Science, 5(6), 713–721. 10.1177/1948550613519682

[bibr12-01461672251391893] Choma B. L. Hodson G. (2017). Right-wing ideology: Positive (and negative) relations to threat. Social Cognition, 35(4), 415–432. 10.1521/soco.2017.35.4.415

[bibr13-01461672251391893] Cohn L. D. Becker B. J. (2003). How meta-analysis increases statistical power. Psychological Methods, 8(3), 243–253. 10.1037/1082-989X.8.3.243 14596489

[bibr14-01461672251391893] Cordano M. Welcomer S. Scherer R. Pradenas L. Parada V. (2010). Understanding cultural differences in the antecedents of pro-environmental behavior: A comparative analysis of business students in the United States and Chile. The Journal of Environmental Education, 41(4), 224–238. 10.1080/00958960903439997

[bibr15-01461672251391893] Corner A. Hahn U. Oaksford M. (2011). The psychological mechanism of the slippery slope argument. Journal of Memory and Language, 64(2), 133–152. 10.1016/j.jml.2010.10.002

[bibr16-01461672251391893] Deppe K. D. Gonzalez F. J. Neiman J. L. Jacobs C. Pahlke J. Smith K. B. Hibbing J. R. (2015). Reflective liberals and intuitive conservatives: A look at the Cognitive Reflection Test and ideology. Judgment and Decision Making, 10(4), 314–331.

[bibr17-01461672251391893] Dittmar H. Dickinson J. (1993). The perceived relationship between the belief in a just world and sociopolitical ideology. Social Justice Research, 6(3), 257–272. 10.1007/BF01054461

[bibr18-01461672251391893] Ditto P. H. Liu B. S. Clark C. J. Wojcik S. P. Chen E. E. Grady R. H. Celniker J. B. Zinger J. F. (2019). At least bias is bipartisan: A meta-analytic comparison of partisan bias in liberals and conservatives. Perspectives on Psychological Science, 14(2), 273–291. 10.1177/1745691617746796 29851554

[bibr19-01461672251391893] Fernbach P. M. Rogers T. Fox C. R. Sloman S. A. (2013). Political extremism is supported by an illusion of understanding. Psychological Science, 24(6), 939–946. 10.1177/0956797612464058 23620547

[bibr20-01461672251391893] Fiagbenu M. E. Proch J. Kessler T. (2021). Of deadly beans and risky stocks: Political ideology and attitude formation via exploration depend on the nature of the attitude stimuli. British Journal of Psychology, 112(1), 342–357. 10.1111/bjop.12430 31724742

[bibr21-01461672251391893] Gampa A. Wojcik S. P. Motyl M. Nosek B. A. Ditto P. H. (2019). (Ideo)logical reasoning: Ideology impairs sound reasoning. Social Psychological and Personality Science, 10(8), 1075–1083. 10.1177/1948550619829059

[bibr22-01461672251391893] Garrett N. Lazzaro S. C. Ariely D. Sharot T. (2016). The brain adapts to dishonesty. Nature Neuroscience, 19(12), 1727–1732. 10.1038/nn.4426 PMC5238933 27775721

[bibr23-01461672251391893] Gerber M. M. Jackson J. (2016). Authority and punishment: On the ideological basis of punitive attitudes towards criminals. Psychiatry, Psychology and Law, 23(1), 113–134. 10.1080/13218719.2015.1034060

[bibr24-01461672251391893] Goh J. X. Hall J. A. Rosenthal R. (2016). Mini meta-analysis of your own studies: Some arguments on why and a primer on how. Social and Personality Psychology Compass, 10(10), 535–549. 10.1111/spc3.12267

[bibr25-01461672251391893] Graham J. Haidt J. Nosek B. A. (2009). Liberals and conservatives rely on different sets of moral foundations. Journal of Personality and Social Psychology, 96(5), 1029–1046. 10.1037/a0015141 19379034

[bibr26-01461672251391893] Guesalaga R. Pitta D. (2014). The importance and formalization of service quality dimensions: A comparison of Chile and the USA. Journal of Consumer Marketing, 31(2), 145–151. 10.1108/JCM-08-2013-0660

[bibr27-01461672251391893] Hafer C. L. Choma B. L. (2009). Belief in a just world, perceived fairness, and justification of the status quo. In Jost J. T. Kay A. C. Thorisdottir H. (Eds.), Social and psychological bases of ideology and system justification (pp. 107–125). Oxford University Press. 10.1093/acprof:oso/9780195320916.003.005

[bibr28-01461672251391893] Haidt J. (2016). Why concepts creep to the left. Psychological Inquiry, 27(1), 40–45. 10.1080/1047840X.2016.1115713

[bibr29-01461672251391893] Haigh M. Wood J. S. Stewart A. J. (2016). Slippery slope arguments imply opposition to change. Memory & Cognition, 44(5), 819–836. https://doi.org/10.3758/s13421-016-0596-9 10.3758/s13421-016-0596-9 26886759

[bibr30-01461672251391893] Haslam N. (2016). Concept creep: Psychology’s expanding concepts of harm and pathology. Psychological Inquiry, 27(1), 1–17. 10.1080/1047840X.2016.1082418

[bibr31-01461672251391893] Haslam N. Dakin B. C. Fabiano F. McGrath M. J. Rhee J. Vylomova E. Weaving M. Wheeler M. A. (2020). Harm inflation: Making sense of concept creep. European Review of Social Psychology, 31(1), 254–286. 10.1080/10463283.2020.1796080

[bibr32-01461672251391893] Haslam N. Vylomova E. Zyphur M. Kashima Y. (2021). The cultural dynamics of concept creep. American Psychologist, 76(6), 1013–1026. 10.1037/amp0000847 34914436

[bibr33-01461672251391893] Hayes A. F. (2018). Introduction to mediation, moderation, and conditional process analysis: A regression-based approach (2nd ed.). Guilford Press.

[bibr34-01461672251391893] Henrich J. Heine S. J. Norenzayan A. (2010). The weirdest people in the world? Behavioral and Brain Sciences, 33(2–3), 61–83. 10.1017/S0140525X0999152X 20550733

[bibr35-01461672251391893] Hibbing J. R. Smith K. B. Alford J. R. (2014). Differences in negativity bias underlie variations in political ideology. Behavioral and Brain Sciences, 37(3), 297–307. 10.1017/S0140525X13001192 24970428

[bibr36-01461672251391893] Inbar Y. Pizarro D. A. Bloom P. (2009). Conservatives are more easily disgusted than liberals. Cognition & Emotion, 23(4), 714–725. 10.1080/02699930802110007

[bibr37-01461672251391893] Jost J. T. (2006). The end of the end of ideology. American Psychologist, 61(7), 651–670. 10.1037/0003-066X.61.7.651 17032067

[bibr38-01461672251391893] Jost J. T. (2017). Ideological asymmetries and the essence of political psychology. Political Psychology, 38(2), 167–208. 10.1111/pops.12407

[bibr39-01461672251391893] Jost J. T. Glaser J. Kruglanski A. W. Sulloway F. J. (2003). Political conservatism as motivated social cognition. Psychological Bulletin, 129(3), 339–375. 10.1037/0033-2909.129.3.339 12784934

[bibr40-01461672251391893] Kahan D. M. (2013). Ideology, motivated reasoning, and cognitive reflection. Judgment and Decision Making, 8(4), 407–424.

[bibr41-01461672251391893] Lakens D. Etz A. J. (2017). Too true to be bad: When sets of studies with significant and nonsignificant findings are probably true. Social Psychological and Personality Science, 8(8), 875–881. 10.1177/1948550617693058 PMC5734376 29276574

[bibr42-01461672251391893] Lamb D. (1988). Down the slippery slope—arguing in applied ethics. Croom Helm.

[bibr43-01461672251391893] Lammers J. Koch A. Conway P. Brandt M. J. (2017). The political domain appears simpler to the politically extreme than to political moderates. Social Psychological and Personality Science, 8(6), 612–622. 10.1177/1948550616678456

[bibr44-01461672251391893] Launis V. (2002). Human gene therapy and the slippery slope argument. Medicine, Health Care and Philosophy, 5(2), 169–179. 10.1023/A:1016052122403 12168992

[bibr45-01461672251391893] Linden S. Panagopoulos C. Azevedo F. Jost J. T. (2021). The paranoid style in American politics revisited: An ideological asymmetry in conspiratorial thinking. Political Psychology, 42(1), 23–51. 10.1111/pops.12681

[bibr46-01461672251391893] Lode E. (1999). Slippery slope arguments and legal reasoning. California Law Review, 87, 1468–1543.

[bibr47-01461672251391893] Mcshane B. B. Böckenholt U. (2017). Single paper meta-analysis: Benefits for study summary, theory-testing, and replicability. Journal of Consumer Research, 43, 1048–1063. 10.1093/jcr/ucw085

[bibr48-01461672251391893] Mooijman M. Hoover J. Lin Y. Ji H. Dehghani M. (2018). Moralization in social networks and the emergence of violence during protests. Nature Human Behaviour, 2(6), 389–396. 10.1038/s41562-018-0353-0 31024159

[bibr49-01461672251391893] Pacini R. Epstein S. (1999). The relation of rational and experiential information processing styles to personality, basic beliefs, and the ratio-bias phenomenon. Journal of Personality and Social Psychology, 76(6), 972–987. 10.1037/0022-3514.76.6.972 10402681

[bibr50-01461672251391893] Palan S. Schitter C. (2018). Prolific.ac—A subject pool for online experiments. Journal of Behavioral and Experimental Finance, 17, 22–27. 10.1016/j.jbef.2017.12.004

[bibr51-01461672251391893] Proferes N. Jones N. Gilbert S. Fiesler C. Zimmer M. (2021). Studying Reddit: A systematic overview of disciplines, approaches, methods, and ethics. Social Media + Society, 7(2), 20563051211019004. 10.1177/20563051211019004

[bibr52-01461672251391893] Rathje S. Mirea D.-M. Sucholutsky I. Marjieh R. Robertson C. Van Bavel J. J. (2023). GPT is an effective tool for multilingual psychological text analysis [Preprint]. PsyArXiv. 10.31234/osf.io/sekf5 PMC11348013 39133853

[bibr53-01461672251391893] Reiljan A. (2020). “Fear and loathing across party lines” (also) in Europe: Affective polarisation in European party systems. European Journal of Political Research, 59(2), 376–396. 10.1111/1475-6765.12351

[bibr54-01461672251391893] Ruisch B. C. Stern C. (2021). The confident conservative: Ideological differences in judgment and decision-making confidence. Journal of Experimental Psychology: General, 150(3), 527–544. 10.1037/xge0000898 32790460

[bibr55-01461672251391893] Schauer F. (1985). Slippery slopes. Harvard Law Review, 99(2), 361. 10.2307/1341127

[bibr56-01461672251391893] Shook N. J. Fazio R. H. (2009). Political ideology, exploration of novel stimuli, and attitude formation. Journal of Experimental Social Psychology, 45(4), 995–998. 10.1016/j.jesp.2009.04.003

[bibr57-01461672251391893] Stenvoll D. (2008). Slippery slopes in political discourse. In Carver T. Pikalo J. (Eds.), Political language and metaphor: Interpreting and changing the world (pp. 28–40). Routledge.

[bibr58-01461672251391893] Sterling J. Jost J. T. Pennycook G. (2016). Are neoliberals more susceptible to bullshit? Judgment and Decision Making, 11(4), 352–360.

[bibr59-01461672251391893] Stern C. West T. V. (2016). Ideological differences in anchoring and adjustment during social inferences. Personality and Social Psychology Bulletin, 42(11), 1466–1479. https://doi.org/10.1177/0146167216664058 10.1177/0146167216664058 27613756

[bibr60-01461672251391893] Svedholm-Häkkinen A. M. Kiikeri M. (2022). Cognitive miserliness in argument literacy? Effects of intuitive and analytic thinking on recognizing fallacies. Judgment and Decision Making, 17(2), 331–361.

[bibr61-01461672251391893] Swami V. Voracek M. Stieger S. Tran U. S. Furnham A. (2014). Analytic thinking reduces belief in conspiracy theories. Cognition, 133(3), 572–585. 10.1016/j.cognition.2014.08.006 25217762

[bibr62-01461672251391893] Talhelm T. Haidt J. Oishi S. Zhang X. Miao F. F. Chen S. (2015). Liberals think more analytically (more “WEIRD”) than conservatives. Personality and Social Psychology Bulletin, 41(2), 250–267. 10.1177/0146167214563672 25540328

[bibr63-01461672251391893] Thelwell M. (2018). Social media analytics for YouTube comments: Potential and limitations. International Journal of Social Research Methodology, 21(3), 303–316. 10.1080/13645579.2017.1381821

[bibr64-01461672251391893] Thomson K. S. Oppenheimer D. M. (2016). Investigating an alternate form of the cognitive reflection test. Judgment and Decision Making, 11(1), 99–113.

[bibr65-01461672251391893] Toner K. Leary M. R. Asher M. W. Jongman-Sereno K. P. (2013). Feeling superior is a bipartisan issue: Extremity (not direction) of political views predicts perceived belief superiority. Psychological Science, 24(12), 2454–2462. 10.1177/0956797613494848 24096379

[bibr66-01461672251391893] van der Burg W. (1991). The slippery slope argument. Ethics, 102(1), 42–65. 10.1086/293369 1463876

[bibr67-01461672251391893] Volokh E. (2003). The mechanisms of the slippery slope. Harvard Law Review, 116(4), 1026–1137. 10.2307/1342743

[bibr68-01461672251391893] Walton D. (2010). Why fallacies appear to be better arguments than they are. Informal Logic, 30(2), 159–184.

[bibr69-01461672251391893] Walton D. (2015). The basic slippery slope argument. Informal Logic, 35(3), 273–311.

[bibr70-01461672251391893] Welsh D. T. Ordóñez L. D. Snyder D. G. Christian M. S. (2015). The slippery slope: How small ethical transgressions pave the way for larger future transgressions. Journal of Applied Psychology, 100(1), 114–127. 10.1037/a0036950 24865577

[bibr71-01461672251391893] Zmigrod L. Rentfrow P. J. Robbins T. W. (2020). The partisan mind: Is extreme political partisanship related to cognitive inflexibility? Journal of Experimental Psychology: General, 149(3), 407–418. 10.1037/xge0000661 31380695

