# Supplementary material for: “And the Next Thing You Know . . .”: Ideological Differences in Slippery Slope Thinking
Source: Pers Soc Psychol Bull. 2025 Nov 13;52(11):3757–72. doi: 10.1177/01461672251391893 (PMC13646407; doi:10.1177/01461672251391893)
Supplement: Supplementary file 1 [file sj-docx-1-psp-10.1177_01461672251391893.docx]

**Supplemental Materials**

**“And the Next Thing You Know…”: Ideological Differences in Slippery Slope Thinking**

**Study 1a Supplemental Analyses**

In a set of exploratory analyses, we conducted a repeated measures ANOVA to test whether the strength of the association between political ideology and slippery slope thinking differed as a function of the specific argument. We found a non-significant interaction between argument and ideology on ratings *F*(5, 193) = 0.46, *p* = .80, *ƞ_p_*^2^ = .01, indicating that the nature and strength of the relation between ideology and slippery slope thinking did not significantly differ as a function of the specific argument. We also examined the pattern of results for each of the six individual slippery slope arguments (see Table S1). We found that the association was significant for five of the six arguments, *p*s < .01. Further, the final argument (which related to whether allowing students to redo one test would eventually lead them to demand to redo *all* tests) was trending in a directionally consistent manner, *p* = .11. These sets of exploratory analyses suggest that the association between conservatism and slippery slope thinking manifests to an equivalent degree across different kinds of arguments, contexts, and content.

**Study 1c Supplemental Analyses**

As in Study 1a, in a series of exploratory analyses we examined whether the pattern of effects differed as a function of the specific vignette (see Table S2). We found a non-significant interaction between ideology and item, *F*(5, 245) = 0.74, *p* = .59, *ƞ_p_*^2^ = .01, indicating that the strength of the relation between ideology and slippery slope thinking did not differ as a function of the specific content or nature of the argument. Further, examining the pattern of effects for each of the individual arguments revealed a significant association for all six of these vignettes (all *p*s < .02).

| Slippery Slope Argument | β |
| --- | --- |
| If we lower the drinking age, next thing we know kids will get to drive at age ten, and vote at fifteen. | .26*** |
| Today late for ten minutes, tomorrow late for an hour, and then someday you will simply cease to show up. | .18* |
| If you break your diet and have one cookie tonight, you will just want to eat 10 cookies tomorrow, and before you know it, you will have gained back the 15 pounds you lost. | .21** |
| If you allow the students to redo this test, they are going to want to redo every assignment for the rest of the year. | .11 |
| You can never give anyone a break. If you do, they'll walk all over you. | .23** |
| If you give in every time your baby cries, he will always pitch a fit to get what he wants, and he will end up in prison because you never set limits. | .21** |

*Table S1*: Standardized beta coefficients between political conservatism and each of the slippery slope arguments from Study 1a. * *p* < .05, ** *p* < .01, *** *p* < .001

| Slippery Slope Argument | β |
| --- | --- |
| If Dan spends that extra $5 today, tomorrow he'll spend an extra $20, and before you know it he'll be completely broke. | .19** |
| If Jim eats that extra 100 calories today, tomorrow he'll eat an extra 500, and before you know it he'll have gained 15 pounds. | .17** |
| If Simon's parents let him skip washing the dishes today, then by next week he’ll stop taking out the trash as well, and by the end of the month, he'll have completely stopped cleaning. | .24*** |
| If we let Mark show up a minute late to work today, he'll be 5 minutes late tomorrow, and before you know it he'll be the last one to arrive. | .20** |
| If John gets a bad score on this test, he'll fail the class. If John fails the class, he's likely to fail out of school completely. | .15* |
| If Bill doesn't keep his living room tidy, he's likely to stop caring about the other rooms as well. If he stops caring about the other rooms, before you know it the whole house will collapse. | .27*** |

*Table S2*: Standardized beta coefficients between political conservatism and each of the slippery slope arguments from Study 1c. * *p* < .05, ** *p* < .01, *** *p* < .001

**Supplemental Study 1**

In Supplemental Study 1, we wished to further assess the robustness of this effect while ruling out a possible alternative explanation for our results—namely, that these effects might have been driven by the targets of the arguments having been men. That is, given that past research has shown that more conservative (versus liberal) individuals tend to hold more gender stereotypical beliefs and expectations (Bock et al., 2017)—which often include the stereotype that men are more likely to engage in risky or reckless behavior (Ellemers, 2018)—it is possible that the results of the previous study were due, in whole or in part, not to ideological differences in slippery slope thinking *per se*, but rather to conservatives believing that men in particular would be more likely to engage in these (often risky or irresponsible) behaviors. To assess this possibility, we conducted a replication and extension of Study 1c. We used the same vignettes from that previous study, but this time randomized the gender of the targets. Gender was indicated by the names of the targets and the pronouns used to describe them.

**Method**

*Participants*

We collected 251 participants from Amazon’s Mechanical Turk. We did not collect demographic information from participants.

*Procedure*

The procedure of this study was identical to that of Study 1c, with the exception of randomly assigning participants to either the *men* condition or the *women* condition. As in previous studies, participants rated how logical they thought each of the six slippery slope arguments was. In the *men* condition, the names used in the statements were traditionally masculine names in the U.S. (Dan, Jim, Simon, Mark, John, and Bill), whereas in the *women* condition, the names were traditionally feminine names in the U.S. (Sarah, Amy, Melissa, Jennifer, Laura, Susan). In addition, the pronouns used in the statements were either masculine (he, him, his) or feminine (she, her, hers). Participants additionally completed the 9-point scale of political ideology as in previous studies (*M* = 4.27, *SD* = 2.32).

**Results and Discussion**

The reliability of participants ratings of these six vignettes was acceptably high (α = .79), and so we averaged them into a single index of slippery slope thinking. We also calculated political extremity in the same manner as previous studies.

Conceptually replicating the results of our previous studies, we once again found that ideology was significantly associated with ratings of the slippery slope arguments, β = .23, *t*(249) = 3.73, *p* < .001, with more conservative individuals rating them as more logical than more liberal individuals. As in previous studies, there was no significant effect of political extremity on slippery slope thinking, β = -.07, *t*(249) = -1.09, *p* = .28.

As anticipated, there was also a main effect of gender of the target on slippery slope judgments, *t*(249) = 2.98, *p* = .003, *d* = .38, such that people rated the arguments as more logical when the actions were performed by men (*M* = 4.37, *SD* = 1.68) than by women (*M* = 3.77, *SD* = 1.46). However, the relation between ideology and ratings of the arguments was not moderated by the gender of the target, β = -.03, *t*(247) = -0.46, *p* = .64. Thus, the association between political conservatism and slippery slope thinking emerged to a similar extent when the arguments regarded both men, β = .24, *t*(133) = 2.88, *p* = .005, and women, β = .20, *t*(115) = 2.19, *p* = .03 (see Figure S1). These results provide further evidence of the robustness of the ideological differences in slippery slope thinking effect, while ruling out ideological differences in gender stereotypes as an explanation for this effect.

*Figure S1.* Scatterplot illustrating the relation between political orientation and ratings of the six slippery slope arguments in Study S1. Participants who judged female targets are represented by green female symbols. Participants who judged male targets are represented by red male symbols. Regression lines illustrate the relations between ideology and slippery slope endorsement for participants in both the female-target condition (in green) and male-target condition (in red). Shaded areas represent the 95% confidence intervals of the regression lines.

**Supplemental Study 2**

The results of the previous studies provided consistent support for the hypothesis that liberals and conservatives differ in the degree to which they endorse slippery slope arguments. Further, these studies also provide evidence that these ideological differences specifically relate to slippery slope thinking in particular (rather than, e.g., general pessimism). Finally, they also demonstrate the robustness of this effect to different stimuli, and rule out a range of alternative explanations for this association. In Study S2, we turned to examining the psychological mechanism behind the conservatism and slippery slope thinking hypothesis.

The psychological mechanisms behind slippery slope thinking have received little attention in past research (although for initial forays into this domain, see Adelman et al., 2021; Corner et al., 2011; Haigh et al., 2016). To our knowledge, no research to date has systematically examined how individual differences in psychological traits and motivations may shape the degree to which an individual endorses or engages in slippery slope thinking. Accordingly, for our first examination of the possible mechanism(s) behind this effect, we cast a wide net. We assessed three potential mechanisms that we derived from past research and theory on the psychological underpinnings of political ideology (e.g., Jost et al., 2003, 2009) and from theory on the nature and structure of slippery slope arguments (e.g., Haigh et al., 2016; van der Burg, 1991; Volokh, 2003; Walton, 2015).

The first account that we tested relates to ideological differences in perceptions of human nature. Specifically, in past research and theory on the nature of political ideology, it has been argued that conservatives hold a more pessimistic view of the human nature, tending to believe that humans are fundamentally bad, immoral, and selfish (e.g., Buck, 2014; de St. Aubin, 1996; Hibbing et al., 2014; Tomkins, 1963). We hypothesized that these beliefs could serve as the causal “engine” behind these chains of negative events—leading conservatives to believe, for example, that relaxing restrictions or self-discipline will lead to increasingly negative behavior and consequences downstream.

The second possible mechanism that we examined was the psychological need for closure. Need for closure refers to a desire or “need” for a clear, decisive answer to a question or problem (Webster & Kruglanski, 1994). Individuals who are higher in the need for closure—including more politically conservative (versus liberal) individuals (Chirumbolo & Leone, 2008; Jost et al., 2003)—tend to dislike ambiguity and uncertainty, and to favor clear-cut responses and solutions to problems. In the context of slippery slope argumentation, this may translate into more “black-and-white” thinking, a desire to decisively resolve the uncertain causal process detailed in the argument, and/or a motivation to interpret the ambiguously negative initial act *A* as more clearly negative in nature, and thus more likely to lead to harmful downstream consequences.

The third and final mechanism that we examined was individual differences in intuitive (versus deliberative) thinking. That is, some research has found that liberals and conservatives differ in the degree to which they rely on intuitive/experiential versus deliberative/rational thinking (Deppe et al., 2015; Sterling et al., 2016; Talhelm et al., 2015; although see Kahan, 2013), with conservatives being more likely to “go with their gut” and provide more intuitive responses, and liberals tending to engage in more extensive deliberation. Given that slippery slope argumentation has been argued to be strongest when it is more intuitively appealing (Corner et al., 2011; Svedholm-Häkkinen & Kiikeri, 2022; Walton, 2010), we hypothesized that ideological differences in intuitive thinking might also help to explain these differences in slippery slope thinking.

To avoid the possibility that responses to our proposed mediator measures might affect ratings of the slippery slope arguments (or vice versa), we conducted this study across two time points. At Time 1, participants rated the slippery slope arguments, and at Time 2 they completed measures assessing our three target mediators.

**Method**

*Participants*

To determine the necessary sample size for this study, we conducted a power analysis for 80% power to detect our hypothesized mediation effect (following the recommendations of, and using the R package created by, Schoemann et al., 2017). Based on the effect sizes observed in Studies 1a and 1c, we anticipated a direct effect (*c*’ path) of .3. Based on our review of past research, we anticipated an *a* path of .2 (i.e., a correlation of .2 between ideology and our perceptions of human nature), and a *b* path of .2 (i.e., a correlation of .2 between perceptions of human nature and slippery slope thinking). We entered these parameters into the model. Consistent with the recommendations of Schoemann et al., 2017, we conducted three separate analyses with 1,000, 5,000, and 10,000 replications, respectively, each with 20,000 monte carlo draws per replication (For researchers wishing to replicate these analyses, set “Random Seed” to 1234). All three analyses converged on virtually identical results (varying less than 1 participant on average), suggesting that the estimates had converged on a stable value. The highest recommended sample was 361 (1,000-replication model), so we set this as our target sample size for this study. We further increased this target sample size to 400 given that we anticipated some attrition between Time 1 and Time 2 of our study. We preregistered this target sample size, whom we recruited from Mechanical Turk. We received 403 complete responses to our Time 1 survey. Three-hundred and forty-seven of these participants (86.1%) returned between 2-7 days later to complete the Time 2 survey (174 women, 173 men; *M*_age_ = 40.42, *SD*_age_ = 12.74). Only participants who completed the Time 2 survey are included in the analyses.

*Procedure*

At Time 1, participants rated 12 slippery slope arguments (the six arguments from Study 1a and the six arguments from Study 1c) on the same 9-point scale from our previous studies. They then provided their political orientation using the same 9-point measure from our previous studies (*M* = 4.51, *SD* = 2.39). At Time 2, participants provided demographic information and completed the scales assessing our three potential target mediators (presented in random order):

*Perceptions of Human Nature*: To assess participants’ views of human nature, we used the philosophies of human nature scale (Wrightsman, 1964). In this scale, participants rate their agreement with statements regarding how most people tend to behave (e.g., “Most people would tell a lie if they could gain by it.” from *1 Strongly disagree* to *7 Strongly agree*). Based on research and theory on the specific dimensions of human nature perceptions that should be expected to differ between liberals and conservatives (de St. Aubin, 1996; Tomkins, 1963), we focused on two dimensions: trustworthiness and altruism. To make the scale comparable in length with our other mediators, we shortened it to 10 (of the original 28) items by selecting the five items from each subscale with the highest item-total correlations from the original research (see our OSF link for exact items). We were interested specifically in whether participants held generally pessimistic beliefs about human nature and therefore reverse-coded items that asked about a positive belief regarding human nature (e.g., “The typical person is sincerely concerned about the problems of others.”).

*Need for Closure*: To assess need for closure, we used the 15-item version of the need for closure scale (Roets & Van Hiel, 2011; Webster & Kruglanski, 1994). The need for closure scale assesses an individual’s self-reported tendency to prefer having a clear answer on a topic compared to confusion or ambiguity. Participants indicated their agreement on a 7-point scale from “Strongly disagree” to “Strongly agree” to statements like “I don’t like situations that are uncertain”.

*Intuitive (Versus Deliberative) Thinking:* We assessed intuitive thinking using the CRT-2 (Thomson & Oppenheimer, 2016; an updated version of Frederick, 2005).^[1]^ The measure consists of four questions that assess an individual’s tendency to override intuitive, but incorrect, responses with more effortful deliberative thinking to arrive at the correct response (e.g., “Emily’s father has three daughters. The first two are named April and May. What is the third daughter’s name?”; intuitive answer: June, correct answer: Emily).

**Results**

The combined twelve-item measure of slippery slope thinking showed strong reliability (α = .88), so we averaged them together to form our index of slippery slope thinking. The need for closure scale (α = .88) and our measure of pessimistic views of human nature (α = .79) also displayed strong reliability. For CRT-2, we summed up the number of incorrect responses to form our measure of intuitive thinking. See Table S1 for zero-order correlations between all measures of interest.

| Variable | 1 | 2 | 3 | 4 | 5 |
| --- | --- | --- | --- | --- | --- |
| 1. Slippery Slope Thinking | − |  |  |  |  |
| 2. Political Orientation | .20*** | − |  |  |  |
| 3. Pessimistic Views of Human Nature | .17** | -.02 | − |  |  |
| 4. Need for Closure | .13* | .10† | .16** | − |  |
| 5. CRT-2 (Intuitive Thinking) | .19*** | .14** | .01 | .10† | − |

*Table S1*. Zero-order correlation table showing the relations between all variables of interest in Study S2. † < .1, * *p* < .05, ** *p* < .01, *** *p* < .001.

Replicating our previous studies, we again found that political ideology was associated with ratings of the slippery slope arguments, with more conservative individuals tending to rate them as more logical than more liberal individuals, β = .20, *t*(345) = 3.86, *p* < .001.

We next examined the relations between our mediator variables and ratings of the slippery slope arguments. We found that all three measures were associated with slippery slope thinking as predicted: intuitive (versus deliberative) thinking, as assessed by the CRT-2, was associated with greater endorsement of slippery slope arguments, β = .19, *t*(345) = 3.63, *p* < .001, as were pessimistic views of human nature, β = .17, *t*(345) = 3.29, *p* = .001, and greater need for closure, β = .13, *t*(345) = 2.47, *p* = .01.

We next examined the associations between ideology and each of our proposed mediators. As predicted, we found that greater political conservatism was significantly associated both with more incorrect (and thus more intuitive) answers on the CRT-2, β = .14, *t*(345) = 2.69, *p* = .008, and marginally associated with greater need for closure, β = .10, *t*(345) = 1.90, *p* = .058. However, contrary to our predictions—and several theoretical perspectives on the nature of ideology (e.g., Hibbing et al., 2014; Tomkins, 1963)—we did not find a significant association between ideology and pessimistic views of human nature, β = -.02, *t*(345) = -0.35, *p* = .73. This suggests that perceptions of human nature do not account for the relation between ideology and slippery slope thinking.

To provide an initial test of whether need for closure and/or intuitive thinking may explain the association between ideology and slippery slope thinking, we next conducted mediation analyses using the PROCESS macro for SPSS (Hayes, 2018). We entered political orientation as the predictor (X) variable and endorsement of the slippery slope arguments as the dependent (Y) measure (Model 4, 10,000 bootstrapped samples). We then ran separate analyses examining each of our mediator variables. We found that only intuitive thinking significantly mediated the relation between ideology and slippery slope thinking (indirect effect: β = .02, 95% CI[.004,.05]); need for cognition was not significant (indirect effect: β = .01, 95% CI[-.002,.03]).

**Discussion**

The results of Study S2 provided initial support for one possible mechanism that may underlie the relation between conservatism and slippery slope thinking: having a more intuitive (versus deliberative) cognitive style. These findings also suggested that two other domains of ideological differences—need for closure and views of human nature—play little-to-no role in explaining this association. More generally, these results also provide insight into the individual difference factors that may predispose an individual towards greater endorsement slippery slope thinking, suggesting that intuition, need for closure, and views of human nature may all play a role.

**Details on the Internal Meta-Analyses**

To focus on the most basic version of these effects, we excluded participants in the *control* conditions from Studies 3 and 5 and the *deliberation* condition from Study 7. We used random-effects meta-analysis models to better generalize beyond the specific samples here and to the general population (Hedges & Vevea, 1998).

**References**

Bock, J., Byrd-Craven, J., & Burkley, M. (2017). The role of sexism in voting in the 2016 presidential election. *Personality and Individual Differences*, *119*, 189–193. https://doi.org/10.1016/j.paid.2017.07.026

Buck, R. (2014). Emotional attachment security as the origin of liberal-conservative differences in vigilance to negative features of the environment. *Behavioral and Brain Sciences*, *37*(3), 308–309. https://doi.org/10.1017/S0140525X13002525

Chirumbolo, A., & Leone, L. (2008). Individual differences in need for closure and voting behaviour. *Personality and Individual Differences*, *44*(5), 1279–1288. https://doi.org/10.1016/j.paid.2007.11.012

Corner, A., Hahn, U., & Oaksford, M. (2011). The psychological mechanism of the slippery slope argument. *Journal of Memory and Language*, *64*(2), 133–152. https://doi.org/10.1016/j.jml.2010.10.002

de St. Aubin, E. (1996). Personal ideology polarity: Its emotional foundation and its manifestation in individual value systems, religiosity, political orientation, and assumptions concerning human nature. *Journal of Personality and Social Psychology*, *71*(1), 152–165. https://doi.org/10.1037/0022-3514.71.1.152

Deppe, K. D., Gonzalez, F. J., Neiman, J. L., Jacobs, C., Pahlke, J., Smith, K. B., & Hibbing, J. R. (2015). Reflective liberals and intuitive conservatives: A look at the Cognitive Reflection Test and ideology. *Judgment and Decision Making*, *10*(4), 314–331.

Ellemers, N. (2018). Gender stereotypes. *Annual Review of Psychology*, *69*(1), 275–298. https://doi.org/10.1146/annurev-psych-122216-011719

Frederick, S. (2005). Cognitive reflection and decision making. *Journal of Economic Perspectives*, *19*(4), 25–42. https://doi.org/10.1257/089533005775196732

Haigh, M., Wood, J. S., & Stewart, A. J. (2016). Slippery slope arguments imply opposition to change. *Memory & Cognition*, *44*(5), 819–836. https://doi.org/10.3758/s13421-016-0596-9

Hayes, A. F. (2018). *Introduction to mediation, moderation, and conditional process analysis: A regression-based approach* (Second edition). Guilford Press.

Hedges, L. V., & Vevea, J. L. (1998). Fixed- and random-effects models in meta-analysis. *Psychological Methods*, *3*(4), 486–504. https://doi.org/10.1037/1082-989X.3.4.486

Hibbing, J. R., Smith, K. B., & Alford, J. R. (2014). Differences in negativity bias underlie variations in political ideology. *Behavioral and Brain Sciences*, *37*(3), 297–307. https://doi.org/10.1017/S0140525X13001192

Jost, J. T., Federico, C. M., & Napier, J. L. (2009). Political ideology: Its structure, functions, and elective affinities. *Annual Review of Psychology*, *60*(1), 307–337. https://doi.org/10.1146/annurev.psych.60.110707.163600

Jost, J. T., Glaser, J., Kruglanski, A. W., & Sulloway, F. J. (2003). Political conservatism as motivated social cognition. *Psychological Bulletin*, *129*(3), 339–375. https://doi.org/10.1037/0033-2909.129.3.339

Kahan, D. M. (2013). Ideology, motivated reasoning, and cognitive reflection. *Judgment and Decision Making*, *8*(4), 407–424.

Pennycook, G., Cheyne, J. A., Koehler, D. J., & Fugelsang, J. A. (2016). Is the cognitive reflection test a measure of both reflection and intuition? *Behavior Research Methods*, *48*(1), 341–348. https://doi.org/10.3758/s13428-015-0576-1

Roets, A., & Van Hiel, A. (2011). Item selection and validation of a brief, 15-item version of the Need for Closure Scale. *Personality and Individual Differences*, *50*(1), 90–94. https://doi.org/10.1016/j.paid.2010.09.004

Schoemann, A. M., Boulton, A. J., & Short, S. D. (2017). Determining power and sample size for simple and complex mediation models. *Social Psychological and Personality Science*, *8*(4), 379–386. https://doi.org/10.1177/1948550617715068

Sterling, J., Jost, J. T., & Pennycook, G. (2016). Are neoliberals more susceptible to bullshit? *Judgment and Decision Making*, *11*(4), 352–360.

Svedholm-Häkkinen, A. M., & Kiikeri, M. (2022). Cognitive miserliness in argument literacy? Effects of intuitive and analytic thinking on recognizing fallacies. *Judgment and Decision Making*, *17*(2), 331–361.

Talhelm, T., Haidt, J., Oishi, S., Zhang, X., Miao, F. F., & Chen, S. (2015). Liberals think more analytically (more “WEIRD”) than conservatives. *Personality and Social Psychology Bulletin*, *41*(2), 250–267. https://doi.org/10.1177/0146167214563672

Thomson, K. S., & Oppenheimer, D. M. (2016). Investigating an alternate form of the cognitive reflection test. *Judgment and Decision Making*, *11*(1), 99–113.

Tomkins, S. (1963). Left and right: A basic dimension of ideology and personality. In R. W. White (Ed.), *The study of lives: Essays on personality in honor of Henry A. Murray.* (pp. 388–411). Atherton Press. https://doi.org/10.1037/12238-017

van der Burg, W. (1991). The slippery slope argument. *Ethics*, *102*(1), 42–65. https://doi.org/10.1086/293369

Volokh, E. (2003). The mechanisms of the slippery slope. *Harvard Law Review*, *116*(4), 1026–1137. https://doi.org/10.2307/1342743

Walton, D. (2010). Why fallacies appear to be better arguments than they are. *Informal Logic*, *30*(2), 159–184.

Walton, D. (2015). The basic slippery slope argument. *Informal Logic*, *35*(3), 273–311.

Webster, D. M., & Kruglanski, A. W. (1994). Individual differences in need for cognitive closure. *Journal of Personality and Social Psychology*, *67*(6), 1049–1062. https://doi.org/10.1037/0022-3514.67.6.1049

Wrightsman, L. S. (1964). Measurement of philosophies of human nature. *Psychological Reports*, *14*(3), 743–751. https://doi.org/10.2466/pr0.1964.14.3.743

1. There is some debate regarding the degree to which the CRT assesses reflection versus intuition, with some arguing that while high CRT scores likely indicate greater deliberation, low scores may not indicate greater intuition (Pennycook et al., 2016). We address this issue in our following study, in which we include separate measures of both deliberation and intuition.
